# Advances Brought by Hydrophilic Ionic Liquids in Fields Involving Pharmaceuticals

**DOI:** 10.3390/ma14216231

**Published:** 2021-10-20

**Authors:** Teresa B. V. Dinis, Francisca A. e Silva, Fani Sousa, Mara G. Freire

**Affiliations:** 1 CICECO—Aveiro Institute of Materials, Department of Chemistry, University of Aveiro, Campus Universitário de Santiago, 3810-193 Aveiro, Portugal; tbdinis@ua.pt (T.B.V.D.); francisca.silva@ua.pt (F.A.eS.); 2 CICS-UBI—Health Sciences Research Centre, Faculty of Health Sciences, University of Beira Interior, Av. Infante D. Henrique, 6201-506 Covilhã, Portugal

**Keywords:** active pharmaceutical ingredients, solubility, concentration, bioavailability, aqueous biphasic systems, ionic liquids

## Abstract

The negligible volatility and high tunable nature of ionic liquids (ILs) have been the main drivers of their investigation in a wide diversity of fields, among which is their application in areas involving pharmaceuticals. Although most literature dealing with ILs is still majorly devoted to hydrophobic ILs, evidence on the potential of hydrophilic ILs have been increasingly provided in the past decade, viz., ILs with improved therapeutic efficiency and bioavailability, ILs with the ability to increase drugs’ aqueous solubility, ILs with enhanced extraction performance for pharmaceuticals when employed in biphasic systems and other techniques, and ILs displaying low eco/cyto/toxicity and beneficial biological activities. Given their relevance, it is here overviewed the applications of hydrophilic ILs in fields involving pharmaceuticals, particularly focusing on achievements and advances witnessed during the last decade. The application of hydrophilic ILs within fields involving pharmaceuticals is here critically discussed according to four categories: (i) to improve pharmaceuticals solubility, envisioning improved bioavailability; (ii) as IL-based drug delivery systems; (iii) as pretreatment techniques to improve analytical methods performance dealing with pharmaceuticals, and (iv) in the recovery and purification of pharmaceuticals using IL-based systems. Key factors in the selection of appropriate ILs are identified. Insights and perspectives to bring renewed and effective solutions involving ILs able to compete with current commercial technologies are finally provided.

## 1. Introduction

The research on more environmentally safer solvents to replace hazardous volatile organic solvents (VOCs) is part of the “Twelve Principles of Green Chemistry” [1]. In this line, ionic liquids (ILs) have been proposed as “greener” alternatives over traditional VOCs. ILs belong to the molten salts category and are composed of a large organic cation and an organic or inorganic anion. Due to the large differences in size and asymmetry in their constituting ions, ILs cannot easily form an ordered crystalline structure and thus may be liquid at temperatures close to room temperature. Accordingly, by general definition, ILs are salts with a melting temperature below 373 K [2]. Despite the relevant properties of most ILs, such as negligible volatility, low flammability, and high thermal and chemical stabilities [2], the possibility of tuning their properties, being therefore designed as “designer solvents”, has been the property at the forefront of most studies [3]. ILs have been used for multidisciplinary purposes, namely in organic chemistry (homogeneous catalysis, Heck reaction, or Suzuki reaction) [4,5,6,7], as new materials (electrolytes for the electrochemical industry and liquid crystals) [8,9,10,11], in biocatalysis (non-denaturing solvents for a diversity of enzymes) [12], in separation processes (of a variety of compounds, including pharmaceuticals) [13], in drugs formulation and drug delivery [14], among many other applications.

**Figure 1:**
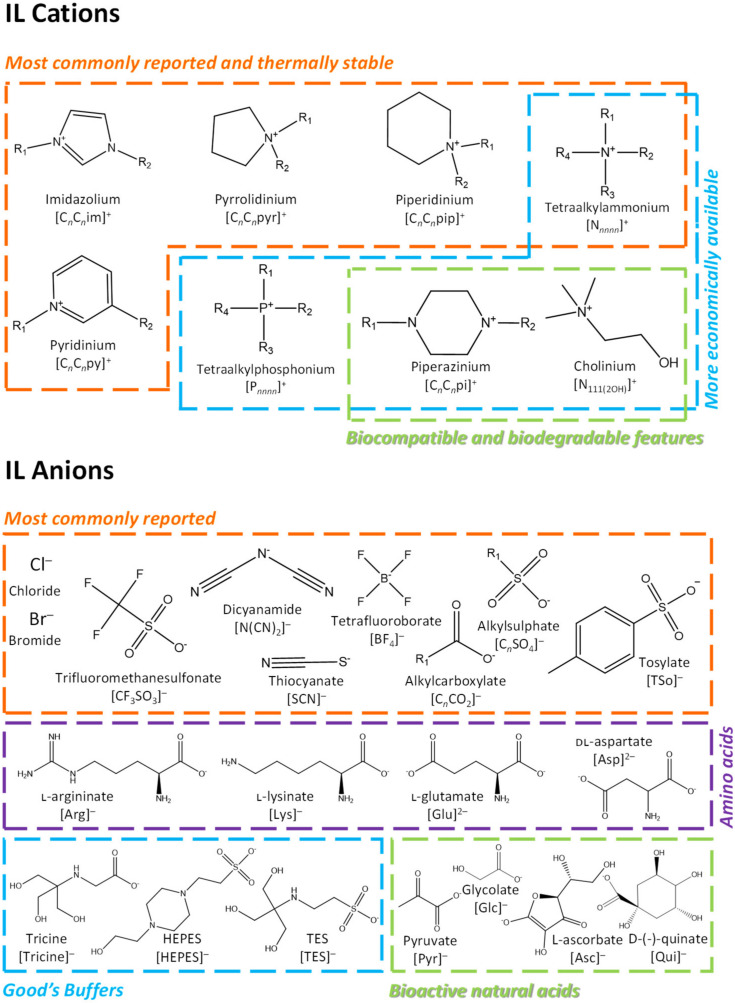
Examples of some chemical structures of IL cations and anions reported in the literature.

Although hydrophobic ILs have been the most investigated (most of them resorting to imidazolium cations paired with fluorinated anions), in the last decade hydrophilic structures for ILs and their mixtures with water have been increasingly investigated as promising alternatives to the widely studied hydrophobic ones. Figure 1 depicts some examples of the chemical structures of IL cations and anions, both comprising ions that would result in hydrophilic or hydrophobic ILs.

In fields related to pharmaceuticals, hydrophilic ILs present several advantages. Hydrophilic ILs are miscible with water and can thus be used as aqueous solutions, thereby providing a more amenable environment, important to maintain the biological activity and structural stability of bioactive compounds [15]. Furthermore, aqueous mixtures would allow for a reduced viscosity and cost reduction of the solvent, while representing more sustainable solvents since the amount of IL used is decreased and water is used [15,16,17]. On the other hand, these ILs, by being water-soluble, can act as co-solvents or hydrotropes to improve the solubility of poorly water-soluble drugs, further allowing the development of enhanced drug-delivery strategies [14]. ILs presenting hydrophilic ions are most of the time cheaper and less eco/cytotoxic than hydrophobic ones, as well as more flexible in design due to the higher number of chemical structures available [18]. Despite these advantages, the high water solubility of hydrophilic ILs may raise some environmental concerns [14]. Therefore, the possible applications of hydrophilic ILs (at both the industrial and academic levels) should focus on chemical structures with lower toxicity and higher biodegradability, combined with low cost, in which bio-based ILs may represent a remarkable option.

Allied to their “designer solvents” nature, the concept “task-specific ILs (TSILs)” has been later adopted by the ILs scientific community. In particular, the possibility of “redesigning” ILs structures exhibiting more benign characteristics have ubiquitously started to be kept in mind, such as by combining the cholinium cation with amino-acid-, biological-buffer- and carboxylic-acid-based anions (Figure 1) [19,20,21,22,23]. Quaternary ammonium- (including cholinium-based) and phosphonium-based ILs (Figure 1) have been reported in the literature as compounds of lower toxicity [24,25] and with a high ability to extract biomolecules [26,27,28]. Although less reported in the literature when compared with the imidazolium- or pyridinium-based counterparts, these cations classes can be produced at a large scale and a lower cost [29,30]. In general, quaternary ammonium-based ILs have demonstrated lower toxicity profiles than their phosphonium-based counterparts and a high ability to stabilize biomolecules [24,31]. It has been demonstrated that cholinium-based ILs provide a good solvation ability and the capacity to stabilize macromolecular native structures, such as proteins [22,32], enzymes [33,34] and nucleic acids [35,36], combined with low toxicity profiles if properly designed [25,37]. Cholinium-based ILs also revealed high biological activity when combined with antioxidant-based anions [38], and an increased solvation ability to selectively separate pharmaceutical products [39,40]. Among quaternary ammonium cations of ILs, significant interest has been demonstrated in analogs of glycine-betaine as well [41,42,43]. In summary, the large range of possible ILs with negligible toxicity and biocompatible features by their proper design supports their use in pharmaceutical applications [44].

In the past decade, a high interest has been noticed in the application of hydrophilic ILs in fields comprising pharmaceuticals, particularly as potential alternatives to the most studied hydrophobic ones. According to the data available in the literature, the application of hydrophilic ILs within fields involving pharmaceuticals can be divided into four categories: (i) to improve pharmaceuticals solubility, envisioning improved bioavailability; (ii) as IL-based drug delivery systems; (iii) as pretreatment techniques to improve analytical methods performance dealing with pharmaceuticals, and (iv) in the recovery and purification of pharmaceuticals using IL-based systems. The main objective of this review is to provide a critical overview of the use of hydrophilic ILs in each one of the described categories, therefore comprising the following sections, followed by a discussion regarding the potential and selection criteria of ILs in these fields. Opposed to the limited range of polarities afforded by VOCs, it is theoretically possible to form at least 1 million different ILs [45], enabling the creation of versatile platforms to enhance solubility, drug delivery, extraction, and purification of pharmaceuticals.

Although there is a large interest in the synthesis of ILs comprising active pharmaceutical ingredients in their ions, it should be remarked that this topic is not the aim of this review, and for which other reviews in the field are recommended [14,46,47].

## 2. ILs Used to Improve the Solubility (Bioavailability) of Pharmaceuticals

**Figure 2:**
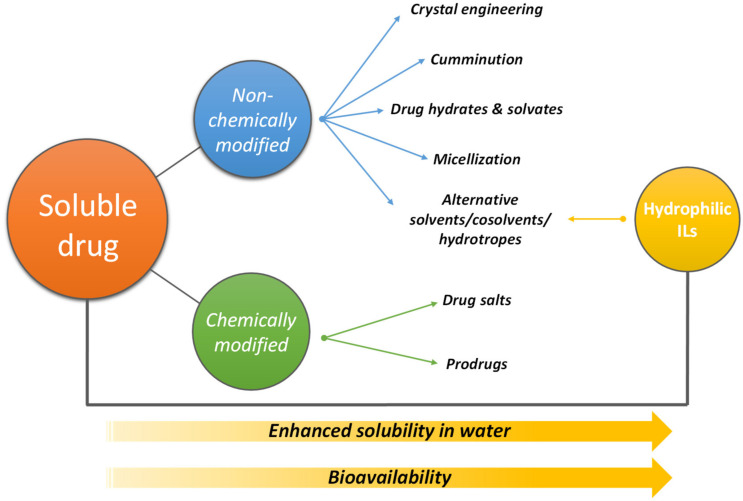
Schematic overview of the main strategies applied to improve the solubility of pharmaceutical compounds, highlighting the main applications of hydrophilic ILs as solvents within the field.

Taking into account their unique solvent abilities, ILs, including those of hydrophilic character and their aqueous solutions, have been investigated as alternative solvents of pharmaceuticals and/or compounds with biological features [46,47,48,49,50]. According to Figure 2, the main strategies generally adopted to improve the solubility of drugs comprise non-chemical (crystal engineering, micellization and use of solvents, co-solvents, and/or hydrotropes) and chemical-based modification processes (such as drugs in form of salts and prodrugs). Figure 2 summarizes the main strategies used to improve the bioavailability of pharmaceutical compounds as well as the several strategies applied in the pharmaceutical arena, further highlighting the use of ILs as solvents, co-solvents, and hydrotropes in the field.

In opposition to approaches where pure ILs are employed as solvents, applications inspired by co-solvency or hydrotropy phenomena resort to the preparation of a mixture, where ILs are added to a major solvent (typically water) [51,52]. Co-solvents are water-soluble compounds with the ability to overcome the limited solubility of hydrophobic solutes in an aqueous solution. Hydrotropes are defined as compounds composed of an amphiphilic part and a hydrophilic part able to largely enhance the solubility of solutes in aqueous solutions; yet, their hydrophobic part is not long enough to be able to form micelles (nor present a critical micellar concentration, CMC) and to behave as surfactants. Co-solvents and hydrotropes diverge on the mechanisms underlying the solubilization process at both the nanoscale and thermodynamic levels [53,54]: (1) in solubilization processes mediated by hydrotropy there is evidence of solute-hydrotrope aggregates formation in solution, with a sigmoidal behavior in solubility being followed in most cases; (2) in solubilization processes mediated by co-solvency there is the solvation of the solute by the mixture formed by water and the co-solvent, with a linear or monotonic solubility trend being followed.

Among the reported works resorting to ILs, several classes of pharmaceuticals and other compounds with biological features have been the target of the investigation, including antioxidants, analgesics, antibiotics, anti-inflammatory, anthelmintic, antidiabetic, antimalarial and anticonvulsant drugs. Table 1 summarizes the main information covered in this section by providing target active pharmaceutical ingredients (and other compounds with biological activity) and the hydrophilic ILs used for their solubilization.

Due to the aqueous environment of the cellular system, hydrophobic or poorly water-soluble drugs tend to form colloidal aggregates, thus interrupting biochemical processes [55,56,57]. Despite this well-known problem, most studies dealing with the solubility of pharmaceuticals and related compounds in IL-based solvation media are focused on hydrophobic (not miscible with water) ILs, which will suffer from the same drawbacks as poorly water-soluble drugs. Even so, relevant studies including both hydrophobic and hydrophilic ILs and respective aqueous solutions have been reported, allowing to appraise relevant molecular-level mechanisms responsible for solubility enhancements. Based on solubility improvements of poorly-water soluble drugs, namely danazol and albendazole, with more hydrophobic ILs, Mizuuchi et al. [58] investigated the potential of ternary mixtures composed of water, a water-immiscible IL, and a water-miscible IL to improve their solubility in water. With this approach, the solubility of albendazole increases by 600-, 2000-, and 4000-fold in equimolar mixtures of water/[C_4_C_1_im][PF_6_]/[C_4_C_1_im][BF_4_], water/[C_4_C_1_im][PF_6_]/[C_6_C_1_im][BF_4_] and water/[C_4_C_1_im][PF_6_]/[C_6_C_1_im]Cl, respectively, when compared with the solubility of the drug in water.

When moving to water-soluble ILs envisioning bioavailability improvements, there are two main approaches: the use of ILs as co-solvents and the use of ILs as hydrotropes. Cláudio et al. [17] demonstrated that several hydrophilic (water-soluble) ILs structures can act as hydrotropes, thus improving the solubility of moderately hydrophobic to hydrophobic compounds in water. In this pioneering work, it was evaluated the hydrotropic behavior of ILs by comparing the solubility of vanillin and gallic acid in aqueous solutions of ILs with that in water, neat ILs, and some common hydrotropes and salting-in inducing salts. Considering the gathered results, the authors highlighted the existence of a synergic effect between water and ILs on the solubility of the two antioxidants, with aqueous solutions of ILs displaying significantly higher ability to dissolve vanillin and gallic acid, with an enhancement up to 40-fold when compared with their solubility in any of the two pure solvents. Experimental data and molecular simulations evidenced that aqueous solutions of the ILs [C_4_C_1_im][SCN] and [C_4_C_1_im][N(CN)_2_] can significantly enhance the solubility of poorly water-soluble compounds through the formation of solute-IL aggregates [17].

Based on the previously discussed results, further works appeared on the investigation of IL hydrotropes to improve the water solubility of active pharmaceutical ingredients (APIs), such as naproxen and ibuprofen [59,60]. In the same line, Sales et al. [61] recently investigated the IL anion effect on the solubility in water of the naturally-occurring drug artemisinin, used in the treatment of malaria. Apart from [C_4_C_1_im][SCN] and [C_4_C_1_im][N(CN)_2_] which were revealed to be the best IL structures capable of improving artemisinin solubility up to 460-fold when compared to the best organic solvents usually employed, the authors established a relationship between the artemisinin solubility and the solvatochromic parameters of the studied ILs, combined with information depicted from the Conductor-like Screening MOdel for Real Solvents (COSMO-RS). Overall, it has been found that the ILs hydrogen-bond accepting ability seems to be a dominant effect behind their hydrotropic capacity to improve the solubility of artemisinin [61].

The discussed results clearly evidence the remarkable capability of ILs to act as hydrotropic agents, where both the IL cation and anion have a solute-dependent influence on their hydrotropic behavior. This behavior will certainly allow one to use the hydrotropic effect to design proper ILs to improve the solubility of target APIs in aqueous media.

**Figure 3:**
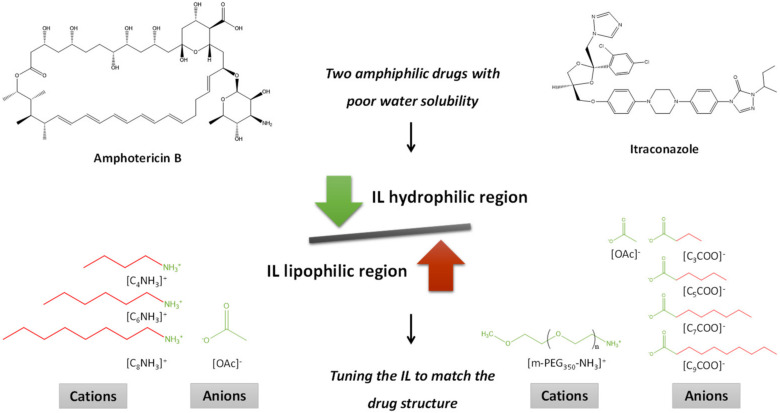
Ionic liquids as tunable solvents to improve the solubility of amphotericin B (cation hydrophobicity tuning) and itraconazole (anion hydrophobicity tuning) in water. Figure adapted from data provided in reference [50].

Focused on the use of ILs as co-solvents, Rogers and coworkers [50] developed a “truly designed IL-excipient” by maximizing interactions of ILs with a targeted drug, essential to simultaneously increase water solubility and prevent drug aggregation. Figure 3 depicts the design of ILs applied as co-solvents to improve the solubility of “hard-case” drugs, namely amphotericin B and itraconazole. A tunable solubility of APIs in water was achieved by playing with the hydrophilic/lipophilic balance in one or both the IL ions. To this end, the authors either manipulated the alkyl side chain length of the [C*_n_*CO_2_]^–^ anion or of the [C*_n_*NH_3_]^+^ cation. In this way, solubility enhancements in water up to 50-fold and 250,000-fold, respectively, were achieved for amphotericin B and itraconazole. Through the proper design of ILs containing functional groups with biodegradable and cheaper characteristics (fatty acid-based anions and polyethylene glycol-based cation), the authors reinforced the potential of real applications of these structures as solubility-enhancers and in drug-based delivery systems. Besides the innovative concept of “truly designed IL-excipient” [50], other works exploring the co-solvency ability of hydrophilic ILs from a thermodynamic perspective are available. For instance, the impact of [C_6_C_1_im]Br on the water solubility of the anticonvulsant drug lamotrigine [62] and the analgesic drug paracetamol [62,63] was appraised in two fundamental studies. The solubility of both drugs was determined as a function of temperature (293.15 to 313.15 K) and IL composition, following an increasing trend with the increase in both conditions. The maximum solubility of lamotrigine was 1.5 × 10^−2^ (in mole fraction at *w*_IL_ = 0.799 and T = 313.2 K), while that of paracetamol was 1.335 × 10^−2^ (in mole fraction at *w*_IL_ = 0.15 and T = 313.15 K) [62,63]. Collected data allowed to determine thermodynamic functions and to correlate the data by activity coefficient models [62,63]. The collection of this kind of information is useful to support the adequate design of ILs as co-solvents as it allows gathering insights on the mechanisms governing the role of ILs as co-solvents as well as the interactions occurring between the IL and the target drug.

In general, the effect of the alkyl side chain of the IL cation was predominantly investigated in works addressing the solubility of APIs in water [17,50,58,61], where an increase in the alkyl side chain length of the IL cation leads to a favorable solubility of more hydrophobic drugs, while for more hydrophilic drugs an opposite trend is observed. In the case of the IL anion [17,61], a more complex phenomenon occurs, although specific API-ILs interactions are considered as driving forces for enhanced dissolution of hydrophobic drugs when compared with their solubility in water. In the past few years, a significant number of studies have risen in the literature reporting the potential as solubility enhancers of ILs comprising ammonium- and phosphonium-based cations [17,64,65,66,67] as well as structures with cheaper and more biodegradable profiles [30,68,69].

Looking for cheaper and more biocompatible alternatives, particular interest has been placed in cholinium-based salts or ILs as APIs solubility enhancers [17,64,70]. Particularly, Heshmeh and coworkers [64] synthesized a new IL structure composed of the cholinium cation and an anion derived from the amino acid tryptophan to improve the aqueous solubility of the poor-water soluble antidiabetic drug glibenclamide. The investigation of the IL concentration effect and the pH in an aqueous medium allowed to achieve improvements of the drug solubility from ca. 290- to 360-fold, respectively. NMR data were used to support the formation of multiple hydrogen bonds and π–π interactions between the IL and the target drug, being responsible for the solubility enhancement of glibenclamide in the presence of the IL.

Supplanting the high incidence of imidazolium-based ILs as hydrotropes in the previously discussed works (e.g., [17,59,60,61]), the use of naturally-derived ILs bearing the cholinium cation and derivatives from phenolic acids as anions were recently proposed [70]. As suggested by Sintra et al. [70], such a type of ILs might cope with the toxicity and efficiency problems exhibited by common hydrotropes, while filling the existing gap of natural hydrotropes. By using gallic, vanillic, and salicylic acids as anion precursors, Sintra et al. [70] reported the water solubility enhancement of two NSAIDs in presence of cholinium-based ILs (viz., [N_111(2OH)_][Gal], [N_111(2OH)_][Van], and [N_111(2OH)_][Sal]). In addition to their biological activities and negligible toxicity features, which are equivalent or even better than their phenolic precursors, all three ILs were able to boost the water solubility of both ibuprofen and naproxen. Solubility enhancements from 500- to 6000-fold were reported, outperforming conventional hydrotropes including urea, sodium tosylate, and sodium benzoate. Moreover, different ILs displayed differently shaped solubility curves, with [N_111(2OH)_][Gal] and [N_111(2OH)_][Van] better suiting applications demanding lower hydrotrope concentrations and [N_111(2OH)_][Sal] performing better when higher hydrotrope concentrations are needed. In this way, it will be possible to take full advantage of the hydrotrope concentration dependence observed with certain IL-API pairs to better de-sign the formulation of the hydrotrope solution to fit the intended application. Despite this hydrotrope concentration-dependent behavior, the best result was achieved when solubilizing ibuprofen in [N_111(2OH)_][Sal] aqueous solutions at 15 mol/kg [70].

Another example of naturally-derived ILs that can enhance the water solubility of sparingly soluble compounds are those bearing L-carnitine as the cation core. Häckl et al. [71] synthesized seven [C*_n_*Car]Br (with 2 ≤ n ≤ 14) ILs to address the ability of their aqueous solutions to solubilize the hydrophobic dye Disperse Red 13 (out of the scope of the present review) and vanillin. After surface tension and dynamic light scattering analyses, it was concluded that these ILs could behave as surfactants depending on the alkyl chain length (i.e., n ≥ 10). Among all [C*_n_*Car]Br tested, [C_6_Car]Br was identified as the IL best solubility enhancer, leading to a ca. 10-fold increase as compared to water (0.62 M at 1 M of IL). When benchmarking the efficiency of [C_6_Car]Br against a conventional hydrotrope (sodium xylene sulfonate, 0.3 M of vanillin solubility at 0.65 M) and two conventional surfactants (dodecyltrimethylammonium bromide, 0.27 M of vanillin solubility at 0.28 M, and cetyltrimethylammonium bromide, 0.13 M of vanillin solubility at 0.08 M), a superior performance was found; as compared to a reference IL ([C_4_C_1_im]Br), the solubility of vanillin was lower although a higher concentration of [C_4_C_1_im]Br was used (0.88 M at 2 M of [C_4_C_1_im]Br versus 0.62 M at 1 M of [C_6_Car]Br). In addition, the authors [71] evaluated the cytotoxicity of [C*_n_*Car]Br ILs, the two reference surfactants, and the imidazolium-based IL against human skin keratinocytes (HaCaT cells). Shorter chain [C*_n_*Car]Br (n ≤ 6) ILs are the least toxic compounds followed by [C_4_C_1_im]Br while remaining [C*_n_*Car]Br ILs being as prone as the two reference surfactants to induce a cytotoxic effect [71]. These results underscore that, if properly designed, ILs are able not only to tackle the toxicity and efficiency challenges faced with typical hydrotropes but also to overcome solubility restrictions associated with common surfactants.

## 3. IL-Based Drug Delivery Systems

**Figure 4:**
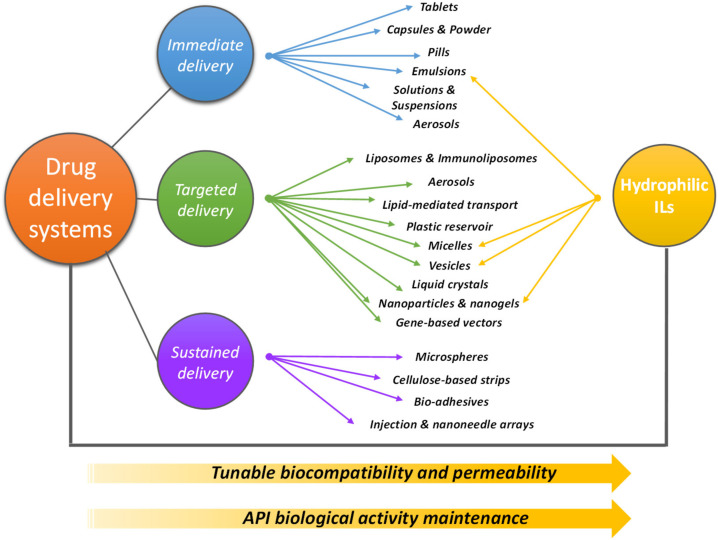
Schematic overview of the main strategies for developing drug delivery systems, highlighting the main applications of hydrophilic ILs as alternative solvents to overcome some of the major limitations associated with the conventional approaches.

The enhancement in the performance of drug delivery processes often relies on the increase of API’s aqueous solubility and resistance to degradation once they are released in the organism [55,57,78]. As summarized in Figure 4, three important delivery approaches have been reported in the literature, which comprises immediate (rapid drugs release, by pills, capsules, suspensions, aerosols), targeted (drug release at higher concentration in specific parts of the organism, by using micelles, vesicles, nanoparticles and nanogels, gene-based vectors) and sustained (drug release at a predetermined rate by maintaining a constant drug concentration for a specific period with minimum side effects, using microspheres, bio-adhesives, (nano)injection arrays) strategies.

In the previous section, it was overviewed the capability of ILs to enhance the solubility of sparingly soluble APIs in water (e.g., [17,50,58,61]). This is the first step for identifying ILs as potential carrier agents in drug delivery devices. Accordingly, a large number of reports comprising ILs as solvents to be employed in the development of promising drug delivery strategies have been published in the literature, namely by IL-based micelles [79,80,81,82], microemulsions [12,48,83,84,85], nanoparticles [86,87,88,89,90], functionalized silica [91,92,93,94], and iongels [95,96,97,98]. These possibilities stand out as the most relevant examples for delivery approaches with ILs, as summarized in Figure 4. Several classes of compounds have been a target of investigation, including antioxidants, analgesics, anticancer drugs, non-steroidal anti-inflammatory drugs, and antibiotics, among others. A summary of the main information under analysis in this section is presented in Table 2, where drug delivery strategies and target active pharmaceutical ingredients (and other compounds with biological activity), as well as the hydrophilic ILs used for their formulation, are detailed. A significant part of these drugs correlates well with those studied in solubility enhancements using ILs, further supporting the connection between both applications.

From IL-containing formulations improving the permeability of particles in controlled and/or topical release processes [82,99,100,101] to the formation of complexes able to release target drugs in pH-sensitive environments [87,89,95,102,103], only the most relevant works will be discussed in this section while highlighting the high chemical versatility of ILs to develop an array of drug delivery systems. Goto and coworkers [104] reported the first IL-based non-aqueous microemulsions as drug delivery devices, where the goal was to improve the transdermal delivery of the poorly water-soluble drug acyclovir. Based on the solvation ability of ILs to dissolve poorly soluble compounds in water and most organic solvents, the authors [104] investigated [C*_n_*C_1_im]-based ILs (*n* = 1, 2, and 4), combined with various anions, namely [BF_4_]^–^, [C_1_CO_2_]^–^, [(CH_3_O)_2_PO_2_]^–^, and [NTf_2_]^–^ to initially investigate the solubility of acyclovir at 298 K. Taking into account the results obtained, acyclovir is only soluble in hydrophilic ILs with anions possessing strong hydrogen-bond acceptors, such as [(CH_3_O)_2_PO_2_]^–^ and [C_1_CO_2_]^–^. Due to the hydrophilic character of the above ILs, no transdermal transport (using Franz diffusion cell with Yucatan hairless micropig skin) of acyclovir was observed having into account the hydrophobic barrier that skin presents, thus requiring other strategies for effective delivery. IL-in-oil microemulsions were then prepared using isopropyl myristate (considered as safe and, therefore, widely used solvent for drug formulation) as the continuous phase, [C_1_C_1_im][(CH_3_O)_2_PO_2_] as the dispersed phase, and Tween-80 and Span-20 as surfactants. The authors [104] suggested that the presence of IL in the micellar core is attributed to electrostatic interactions between the positively charged imidazolium cation of IL and the electronegative oxygen atoms oxyethylene units of Tween-80, whereas Span-20 was found to facilitate microemulsion formation. Skin permeability to acyclovir was increased several orders of magnitude when compared to results obtained for the cream currently employed on the market. The combined effects of hydrophilic IL and lipophilic components of IL-in-oil microemulsions thus enhanced the skin permeability to acyclovir. Despite these promising results, the authors highlighted the need for further studies to evaluate the co-contribution of the IL on improving the transport of the pharmaceutical compound through the skin.

Other drug molecules, namely methotrexate and dantrolene, were studied in two subsequent articles [47,48], in which other important parameters have been evaluated, such as emulsion properties, phase diagrams, droplet diameter, emulsion stability, drug uptake, and cytotoxicity. By using the same aforementioned set of IL structures, the authors confirmed that more hydrophilic ILs can create microemulsions with the remaining components in the system, whose tendency was correlated with the solubility of the ILs in water [47]. Compared with IL-free systems, it was observed a significant improvement in the solubility of the APIs methotrexate and dantrolene in IL-based microemulsions, which was justified by the existence of hydrogen bonding occurring between the IL anions and the polar groups of the drugs. Furthermore, cytotoxicity assays performed on the reconstructed human epidermal model showed that 80% of cell viability is attained with the microemulsion containing 4% of [C_1_C_1_im][(CH_3_O)_2_PO_2_] as the dispersed phase compared with the control experiment (D-PBS) [48]. On the other hand, Dobler et al. [105] reported the influence of imidazolium-based ILs on the formulation of water-in-oil and oil-in-water microemulsions. Through the use of a hydrophilic IL, [C_6_C_1_im]Cl, and a hydrophobic IL, [C_4_C_1_im][PF_6_], the following parameters were evaluated: particle size distribution, rheological properties, pH, emulsion stability, drug uptake, and cytotoxicity. Interestingly, owing to its specific properties and depending on the water content, [C_6_C_1_im]Cl can replace different phases of the emulsion: it can be used as the oil [106] or the water [107] phase. Cytotoxicity evaluation was obtained for both ILs using human keratinocyte (HaCaT) cell lines [105]. The values of EC_50_ (the effective concentration resulting in 50% reduction of processes) correspond to 5 and 7 mmol/L for [C_6_C_1_im]Cl and [C_4_C_1_im][PF_6_], respectively.

Recently, it has been reported that micelles can act as excellent media for the encapsulation of hydrophobic molecules [108,109,110,111,112]. In particular, surface-active ILs (SAILs) can form micelles in an aqueous solution above the CMC and they have been reported as more effective systems to form micelles over conventional surfactants [113]. Pal et al. [114] studied the potential of SAILs, namely [C_12_C_1_im]Cl and [C_14_C_1_im]Cl, for the delivery of lidocaine hydrochloride. The interactions between the drug and SAILs were appraised by studying the micelles’ aggregation behavior using surface tension, conductance, and fluorescence techniques. It was demonstrated that lidocaine hydrochloride has a pivotal role in modulating the aggregation properties of SAILs; the drug molecules absorbed on the surface of the aggregates decrease the electrostatic repulsions among head groups, thereby decreasing aggregation. The results gathered revealed a high potential of SAILs to develop more efficient drug delivery processes. In the same line, Dandpat et al. [81] investigated the aggregation and dissociation behavior of hydrophobic drugs, namely rutaecarpine, in the presence of [C_12_C_1_im]Br by using spectroscopic, microscopic, and crystallographic analysis. The authors found that drug aggregation decreases in solution with the gradual addition of [C_12_C_1_im]Br. This work revealed important facts about the capability of SAILs on dissociating and modulating the morphology of colloidal aggregates to monomers, which play a relevant role in enhancing the biological activity of hydrophobic drugs in cellular systems.

Viau et al. [96] worked on the development of shaped iongels containing ILs as drug delivery devices of ibuprofen. The authors synthetized [C_4_C_1_im][Ibu], by considering that ibuprofen is insoluble in most ILs. Then, [C_4_C_1_im][Ibu] was used to synthesize iongels by silica sol-gel processing using pure tetramethoxysiloxane or tetramethoxysiloxane/methyltrimethoxysilane mixtures of 75/25 or 50/50 mole fractions at acidic conditions. The thermogravimetric analysis demonstrated a high loading of the Ibu-based IL incorporated in the iongels, with 0.8 g ibuprofen per g of dried silica. Furthermore, release kinetics were found to be slower with iongels compared with crystalline ibuprofen and pure [C_4_C_1_im][Ibu], thus demonstrating the capability of these novel systems as reservoirs for the controlled delivery of ibuprofen.

Tuning the solubility of APIs in IL-aqueous systems while modulating the aggregation of drug molecules is a relevant topic when developing drug delivery strategies [115]. Nevertheless, the bioavailability of ILs in aqueous systems must also have to be considered and carefully analyzed when designing the IL structure for the targeted delivery. The solubility and interactions with water do not only prevail for an effective delivery of APIs but also determine the biological profile of ILs, which further depend on their hydration state. Ohno et al. [116] established a threshold hydration number: ca. seven water molecules per ion were suggested to be the boundary at which biological effects of IL-water mixtures change significantly. The authors [116] verified that water activity, which is an important factor to sustain microbial activity, is directly related to the hydration number in IL-water mixtures.

**Figure 5:**
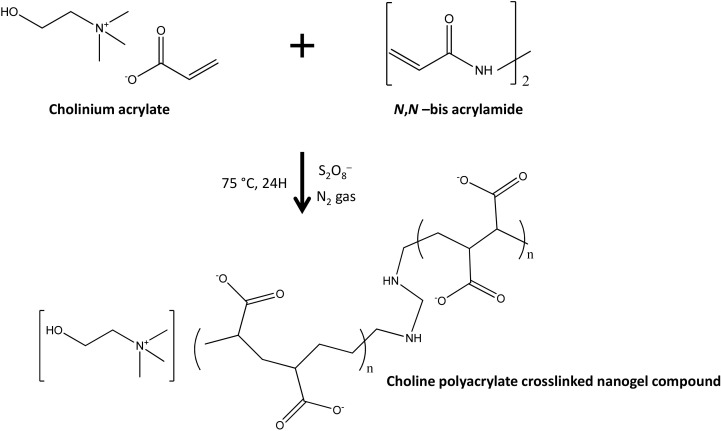
Synthesis of choline polyacrylate-based crosslinked nanogels by emulsion polymerization in cyclohexane and water mixture. Figure adapted from data provided in reference [95].

Zakrewsky et al. [99] reported, for the first time, the combined use of ILs with antimicrobial behavior and low cytotoxicity to cope with biofilm-protected microbial infections and to enhance transdermal delivery. The authors studied cholinium-based ILs, with [N_111(2OH)_][Ger] identified as the best candidate, namely with excellent antimicrobial, minimal cytotoxicity, and effective permeation enhancement for drug delivery. Regarding transdermal delivery applications, a 16-fold enhancement of cefadroxil delivery was observed with a formulation containing [N_111(2OH)_][Ger] in comparison to an aqueous solution. In addition, in vivo tests were performed for [N_111(2OH)_][Ger] by using a biofilm-infected wound model, with >95% bacterial death observed after 2 h of treatment and no skin irritation detected. Prasad and coworkers [95] reported the synthesis of stimuli-responsive nanogels for long-term release towards the simultaneous polymerization and crosslinking of a polymerizable biobased IL (PIL) (Figure 5). A prolonged release of the anticancer drug 5-fluorouracil at pH = 1.2 was observed during 10 days at the human body temperature. Moreover, no substantial drug release was observed at pH = 5 and 7.4, being this novel class of nanogels promising candidates in the formulation of pH-sensitive systems for in vivo release of specific therapeutic agents. Nevertheless, the preclinical application and scale-up of these “smart materials” are issues that still need to be accomplished towards their real application.

## 4. Pretreatment/Concentration of Pharmaceuticals to Improve Analytical Analysis

**Figure 6:**
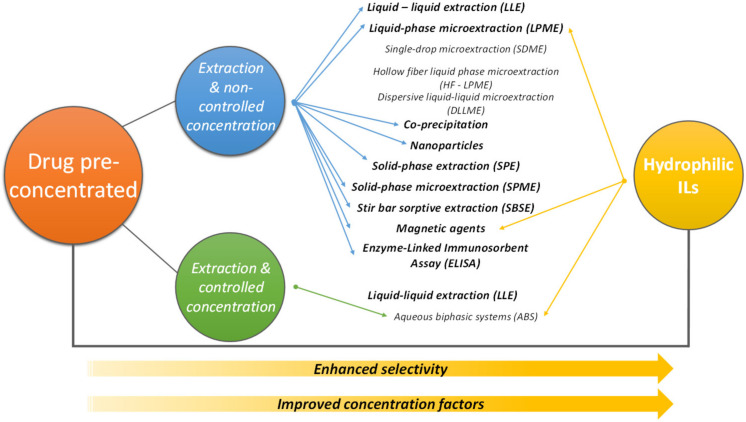
Schematic overview of the main strategies for developing pre-treatment strategies of samples comprising pharmaceuticals, highlighting the main applications of hydrophilic ILs as alternative solvents to overcome the main limitations associated with current approaches.

The application of ILs in pharmaceutical areas has not only been found in the solubilization of drugs and development of drug delivery systems, but also in many fields requiring chemical analysis [14,117,118,119,120] Because of their unique and flexible properties, ILs have been applied in the pretreatment of samples to improve analytical analysis performance, including the monitoring of APIs in the environment and to improve diagnosis and doping control. As summarized in Figure 6, pre-concentration approaches for pharmaceuticals can be mainly distinguished according to their ability/purpose to control the final concentration and purity degree to avoid interferences.

As ILs can be designed to have preferential affinity for a target analyte, they may allow one to overcome the main limitations present in conventional sample pretreatment techniques with controlled concentration approaches, as depicted in Figure 6. For example, it has been demonstrated that the inclusion of polar groups in an IL can promote dipolar interactions between the IL and polar analytes [119]. On the other hand, dispersive interactions with nonpolar analytes are promoted by choosing ILs with long aliphatic alkyl chains. Therefore, ILs allow adequate tailoring in selectivity allowing to reduce interferences, and due to their large solvation potential to simultaneously extract and concentrate the target analyte.

The application of ILs in sample pretreatment and concentration techniques is a well-explored topic of research. ILs can be applied as solvents, additives, or even as functional materials in solvent- and sorbent-based extractions and their miniaturized variants, namely liquid-phase microextraction (LPME) and solid-phase microextraction (SPME) [121]. However, in solvent-based (micro)extraction, hydrophobic ILs are most often applied due to their immiscibility with the samples to be analyzed (mainly of aqueous nature). Given the lower degree of tunability and limited aptitude of hydrophobic ILs to extract polar compounds when compared to their hydrophilic counterparts, strategies enabling the use of hydrophilic ILs in solvent-based extraction should be pursued. In this domain, aqueous biphasic systems (ABS), in which an additional phase-forming component dissolved in water is added to the hydrophilic IL to induce phase separation (being at least ternary systems), are seen as suitable approaches [122]. In addition to their application in ABS, there are other uses of hydrophilic ILs as solvents holding promise in the field of sample pretreatment [121,123,124]: (1) in LPME involving oil samples; (2) as dispersing agent to revisit IL-based dispersive liquid-liquid microextraction (IL-DLLME), and (3) in in situ IL-DLLME.

This section provides an overview of recent and relevant works on the implementation of hydrophilic ILs as extraction solvents in fields related to analytical chemistry for the analysis of pharmaceuticals. Focus is mainly placed on ABS, which allows concentration factors (CFs) to be more easily adjusted to the final application, but applications involving DLLME are also mentioned (Figure 6). Table 3 overviews the active pharmaceutical ingredients (and other compounds with biological activity) under analysis in this section along with the ILs and the pretreatment/concentration approach applied. With antibiotics corresponding to one of the dominant pharmaceutical classes studied, it should be also emphasized the need to detect and quantify the presence of antibiotics in the aquatic environment as one of the most important warmings on the expected dangerous development of multi-resistant organisms in decades ahead.

### 4.1. IL-Based ABS

The first LLE study involving ILs proposed in the literature comprised water-immiscible ILs, i.e., hydrophobic ILs with low extraction potential for polar compounds from water. The appearance of the first LLE system formed by water-miscible ILs and salting-out salt species, denoted as aqueous biphasic systems (ABS), has been recognized as a solution to this problem, being pioneeringly demonstrated by Rogers and coworkers [125]. In the following decades, ABS composed of ILs and a myriad of other phase-forming components have been investigated (e.g., polymers, amino acids, carbohydrates, surfactants, and polar organic solvents), including their application in the pre-treatment of several samples targeting a more accurate identification/quantification of different compounds [126,127,128,129]. In this field, IL-based ABS offer several advantages over typical polymer-based ones and IL-water biphasic systems. These advantages compile low viscosities and/or a wider hydrophilic-hydrophobic range for tailored extraction efficiencies due to the large range of water-miscible ILs available and remaining phase-forming components that can be combined in different mixture compositions. Moreover, these systems may enable a faster and easier phase separation and, consequently, phase volume ratio (i.e., CFs) manipulations can be easily attained [126,129,130,131,132]. Parameters such as pH, IL content and salt content, as well as IL and salt type have been widely reported as important factors influencing the extraction efficiency and concentration ability of pharmaceutical compounds [126,127,128,129].

Within the field of sample pretreatment, ABS composed of ILs and salts (either organic or inorganic) are likely the most studied. The main reasons behind this trend are: (1) the strong salting-out aptitude displayed by some salts that may induce the complete extraction of the target analyte(s) to the IL-rich phase, contributing to more accurate and precise analytical results; (2) by properly selecting the IL-salt pair, wider biphasic regimes are usually provided, enabling a wider array of workable tie-lines (TLs) and, consequently, to better select a target and achievable CF. Two seminal works [132,133] published during the first decade of the 2000s reported on the use of IL-salt-based ABS for the pretreatment of human fluids. By applying the widely studied IL [C_4_C_1_im]Cl in combination with phosphate salts, Liu and coworkers [132] and Du et al. [133] reported CFs of 10 and 20 for anabolic androgenic steroids (testosterone and epitestosterone) and proteins (serum albumin as biomarker), respectively, from human fluids. However, the complete characterization of the ABS phase diagrams, determination of the respective TLs, and mass balance approaches, allowed the application of ABS composed of ILs and inorganic salts to reach higher CFs. The principle behind the use of ABS for the simultaneous extraction and concentration of a target analyte using an ABS or ternary system relies on the fact that the CF can be manipulated through the composition of the initial mixture along a given TL. Each TL describes the composition of each phase under equilibrium for a given initial mixture composition of the ABS. The decrease of the extractive phase volume leads to an increase in the CF of the analyte. The concentration procedure is satisfied by two fundamental requisites: (1) to find an ABS with a TL capable of leading to the complete extraction and without the saturation of the extractive phase, and (2) to use a long TL (or with a proper length) to achieve CFs as high as possible.

Passos et al. [129] reported the application of ABS composed of a wide number of hydrophilic ILs, namely [C_2_C_1_im]Cl, [C_4_C_1_im]Cl, [C_6_C_1_im]Cl, [C_1_C_1_im]Cl, [C_4_C_1_pyr]Cl, [N_4444_]Cl, [P_4444_]Cl, and [N_111(2OH)_]Cl, and the inorganic salt K_3_PO_4_, to completely extract and concentrate up to 100-fold the endocrine disruptor bisphenol A (BPA) from biological fluids, aiming its monitoring in the environment and impact in human health. When dealing with pharmaceutical contaminants in environmental aqueous samples, it is necessary to have even higher CFs due to their presence at vestigial concentrations. With this goal in mind, Dinis et al. [126] developed a concentration technology based on IL-based ABS capable to predict experimental CFs having into account the concentration levels at which the pharmaceutical tracers could be present in real aqueous samples. Several hydrophilic ILs, namely [C_2_C_1_im][N(CN)_2_], [C_4_C_1_im]Br, [C_4_C_1_im][N(CN)_2_], [C_4_C_1_im][SCN], [C_4_C_1_im][CF_3_SO_3_], [C_4_C_1_im][TsO], [C_4_C_1_im][CF_3_CO_2_], [C_6_C_1_im][N(CN)_2_], [N_4444_]Cl, and [P_4444_]Cl were initially studied for ABS formation with the organic salt KNaC_4_H_4_O_6_, followed by the optimization of the extraction efficiency of the synthetic hormone 17*α*-ethinylestradiol (EE2). Through the selection of the most promising ABS composed of [C_4_C_1_im][N(CN)_2_] and KNaC_4_H_4_O_6_, which allows the complete extraction of EE2, the authors were able to concentrate the pharmaceutical from aqueous samples with a CF up to 1000-fold, further allowing the adequate detection and quantification of EE2 in wastewater samples through HPLC-FD. In all these investigations, no major interferences of the ILs as phase-forming components have been found with the analytical equipment and respective signals. Although remarkable results have been reported with IL-based ABS, these works [126,129] only address the extraction and concentration of individual compounds. Yet, when dealing with complex matrices, such as wastewater samples, the presence of several pollution tracers has to be considered. Overall, IL-based ABS have been successfully reported as promising strategies for the extraction and controlled concentration of analytes (Figure 6), which is due to the high extraction efficiencies and tailoring ability afforded by ILs as phase-forming components.

Further diversifying the range of human fluids that can be pretreated by IL-salt-based ABS, Flieger and Czajkowska-Zelazko [134] employed systems bearing [C_4_C_1_im]Cl and K_3_PO_4_ or K_2_HPO_4_ to extract the antimalarial drug quinine from human plasma. After optimizing the IL composition as well as the salt type and composition, the use of K_2_HPO_4_ was more efficient in the extraction of quinine. The quantification of quinine in a plasma sample from a healthy subject after consumption of tonic water was achieved by injecting the IL-rich phase in an HPLC system with fluorescence detection, detecting values as low as ca. 2.5 µg/mL [134].

In an attempt to go further on the potential of IL-based ABS as pre-treatment strategies of a wider range of pollution tracers, Freire’s group [127,135] reported additional studies comprising the application of IL-based ABS for the simultaneous extraction and concentration of different groups of pharmaceutical tracers. Important parameters were investigated, namely extraction efficiencies, pH of the coexisting phases, and sample preparation conditions. Another important parameter is the solubility of the pharmaceutical tracers in the extractive phase, i.e., the IL-rich phase, which enables one to confirm the amount of pharmaceutical tracers that can be simultaneously extracted without saturating the IL-rich phase. Solubility determinations allowed to estimate the maximum allowable CF of pharmaceutical tracers in the IL-rich phase, which mainly depends on solute physical-chemical properties and IL structure. Dinis et al. [127] investigated ABS composed of several hydrophilic ILs and the organic salt K_3_C_6_H_5_O_7_ for the simultaneous extraction and concentration of two well-known human pollution tracers, namely caffeine (CAF) and carbamazepine (CBZ). The ILs [C_4_C_1_im]Cl, [C_4_C_1_im]Br, [C_4_C_1_im][N(CN)_2_], [C_4_C_1_im][SCN], [C_4_C_1_im][CF_3_SO_3_], [C_4_C_1_C_1_im]Cl, [C_4_C_1_pip]Cl, [C_4_C_1_pyr]Cl, [N_4444_]Cl, and [P_4444_]Cl were investigate to optimize the simultaneous extraction of caffeine and carbamazepine, resulting in extraction efficiencies ranging between 95 and 100% towards the IL-rich phase in a single-step. The system composed of [N_4444_]Cl revealed the highest extraction efficiency for both compounds; therefore, it was further applied to pretreat real wastewater effluent samples, with recovery results of 90 ± 16% for CAF and 87 ± 9% for CBZ. The CFs achieved allowed the use of HPLC-UV to quantify both pharmaceuticals. By the determination of the saturation solubility values of CAF and CBZ in the IL-rich phase (28.60 ± 0.27 g·L^−1^ and 8.26 ± 0.60 g·L^−1^, respectively), estimated and “ideal” CFs of up to 28,595-fold for CAF and up to 8259-fold for CBZ were proposed, i.e., up to the saturation of the IL-rich phase. In a subsequent study, Almeida et al. [135] reported the simultaneous extraction and pre-concentration of three antibiotics from the fluoroquinolones family (FQs), namely ciprofloxacin, enrofloxacin, and norfloxacin, and three non-steroidal anti-inflammatory drugs (NSAIDs), namely diclofenac, naproxen, and ketoprofen. Several ILs were screened, resulting in optimized extraction efficiencies up to 98% for FQs and up to 100% for NSAIDs. The system composed of [N_4444_]Cl was selected to be further validated to treat real wastewater effluent, allowing the identification and quantification of ciprofloxacin and diclofenac by HPLC-UV. To the best of our knowledge, no extremely high CFs have been achieved with weak salting-out species. This is due to the strong salting-out effect required to promote the enrichment of the target species in the IL-rich phase, allowing also to have phase diagrams with a larger biphasic region and thus able to provide a long TL length and high CFs.

So far, examples of IL-salt-based ABS as pretreatment techniques of human fluids and wastewater treatment plants (WWTPs) effluents were given [126,127,129,132,133,134,135]. It should be however highlighted that these types of ABS can also be applied to analyze pharmaceuticals present in other types of samples, including aqueous extracts of traditional Chinese herbs [136], river/lake/pond/ground/feed waters [137,138,139,140], food (e.g., honey and milk) [139] and pharmaceutical dosage forms [141] (Table 3). A predilection towards phosphate salts (e.g., K_3_PO_4_, K_2_HPO_4_) is clear in most works [129,132,133,134,136,137,138], likely due to their strong salting-out aptitude, which enables efficient extractions and selecting long TLs. Still, it should be underlined that other salt options including carbonate- [137,141], citrate- [127,135,139], tartrate- [126] and malate-based [140] salts have been reported with success. This fact further underpins the possibility of finely tuning the chemical structure of ABS constituents (not only that of the IL) towards the intended application. Furthermore, and given that one of the main advantages of IL-based ABS is their versatile formulation, components other than salts can be used. In this context, non-ionic surfactants and sodium dodecyl sulfate were added to IL-salt formulations to form ABS and further preconcentrate antibiotics contained in food or biological samples prior to analysis by HPLC with a UV detector [142,143].

Useful technological improvements in sample pretreatment and preconcentration approaches can be additionally brought about by using magnetic ILs [144]. Magnetic ILs hold potential in the field due to the possibility of joining the flexible physicochemical properties of ILs with those of magnetic compounds. By responding to external magnetic fields, magnetic ILs provide a more expedite and efficient phase separation and collection, while streamlining the recovery and reuse of the IL [144]. Hydrophobic magnetic ILs in LPME have been widely reported [144], but pretreatment strategies resorting to ABS can take as well full advantage of magnetic ILs features. Examples of hydrophilic magnetic ILs used in the formulation of ABS targeting the analysis of pharmaceuticals entail those bearing the magnetic anion [TEMPO-OSO_3_]^–^ and the organic cations [C_1_C_1_C_1_C_1_guan]^+^ [145] and [N_115(2OH)_]^+^ [146]. These ILs combined with K_3_PO_4_ allowed preconcentrating chloramphenicol from environmental water samples (CF = 147.2) [145] and berberine hydrochloride from herbal extracts (CF = 127.7) [146], enabling quantification by HPLC-UV.

In summary, the area of research of hydrophilic ILs as ABS components within the (bio)analytical field is currently dominated by imidazolium-based ILs [126,127,129,132,133,134,135,136,137,138,139,142,143] with the emergence of some works dealing with quaternary ammonium-, guanidinium-, and phosphonium-based compounds [126,127,129,135,141,145,146]. Contrarily to the application of ILs in pharmaceuticals downstream or the development of new drug delivery devices, less attention has been given to toxicological, cytotoxicological, and biodegradability effects of ILs in the analytical field. This is also because only small amounts of ILs are required for pre-concentration routes [126,129] in the sense that the IL-phase is where the target analyte is enriched, being the phase with a significantly lower volume. Nevertheless, these features should be addressed as well while focusing on the goals of Green Analytical Chemistry [147].

### 4.2. Other Techniques

As mentioned above, the use of hydrophilic ILs in sample pretreatment strategies other than ABS includes, for instance, DLLME techniques (e.g., as extraction solvent, as dispersing agent, and in in situ IL-DLLME) [121,123,124]. Since hydrophilic ILs are miscible with aqueous samples, they are not adequate to most LPME applications; yet, it is possible to play with their immiscibility with oil samples to create novel pretreatment techniques [148,149]. In this domain, Zhu et al. [149] proposed the use of hydrophilic magnetic ILs ([C*_n_*C_1_im][FeCl_4_], with *n* = 4, 6, and 8) in DLLME to extract endocrine disruptors (BPA and 4-nonylphenol) from vegetable oils prior to HPLC-MS/MS. In the developed method, acetone served as a dispersant to improve the dispersion of the IL within the sample. After assessing of impact of several operating factors (e.g., IL amount, time, dispersant amount), [C_6_C_1_im][FeCl_4_] was deemed the most efficient solvent and the optimal DLLME conditions were identified. The analytical method developed exhibited good precision (relative standard deviations lower than 4.1%) and limits of detection of 0.1 and 0.06 µg/kg for bisphenol A and 4-nitrophenol, respectively. Despite achieving equivalent to lower limits of detection than other previously reported methods, two technical challenges were encountered: (1) the impossibility of directly injecting the IL phase in the HPLC equipment due to viscosity issues, making a back-extraction step using *p*-xylene mandatory and (2) the need to add Fe_3_O_4_ nanoparticles and a centrifugation step to assist the magnetic separation due to limited magnetic properties of [C_6_C_1_im][FeCl_4_]. In other work involving vegetable oils and the analysis of herbicides with the same magnetic IL, similar technical challenges were faced and solved by the addition of carbonyl iron powder (to speed up the phase separation) and of ethyl acetate as the back-extraction solvent [148].

Conventionally, IL-DLLME is carried out by using the hydrophobic IL as the extraction solvent and a dispersing agent, which is soluble in both aqueous solution and the hydrophobic IL and most often a polar organic solvent (e.g., methanol, acetone) [124]. To replace volatile organic solvents, ILs may also find application as a dispersing agent as shown by Wang et al. [150], who designed an IL-DLLME method for the preconcentration of triclosan and methyl triclosan from human fluids prior to HPLC-DAD analysis. In an ultrasound-assisted method, hydrophobic ILs bearing the [PF_6_]^-^ anion and a mixture of [C_4_C_1_im][BF_4_] and [C_4_C_1_im][NPA] ILs were used as extraction and dispersing solvents, respectively. The incorporation of [C_4_C_1_im][NPA] in the dispersant mixture provided higher extraction efficiency of the target analytes and controlled the pH while expediting the entire extraction process (as compared to common organic solvents or [C*_n_*C_1_im][BF_4_] ILs) and assuring compatibility with the analytical equipment. While accurate and precise results were obtained, the proposed method led to lower or similar limits of detection (0.11 µg/L for triclosan) when compared with previous methods based on SPE and LC-MS/MS detection (0.1 to 0.9 µg/L) in a quicker way (18.4 min versus >260 min in the pretreatment stage) [150]. Following the same approach, the same authors [151] more recently transposed the advantages of using a mixture of [C*_n_*C_1_im][BF_4_] and [C_4_C_1_im][NPA] as a dispersing agent in IL-DLLME to the preconcentration of trace tetracycline-based antibiotics from food matrices.

In situ IL-DLLME is a sample pretreatment technique, whereupon the addition of an anion-exchange reagent to an aqueous solution containing a hydrophilic IL, a metathesis reaction takes place. At this point, a hydrophobic IL capable of being dispersed through the sample is yielded, producing microdroplets where the target analytes are enriched [121]. Reports on the determination of antibiotics [152], phenolic endocrine-disrupting chemicals [153,154] and triazole fungicides [155] in a multitude of samples (viz., food, environmental water samples, toys, pacifiers, industrial effluents) with the aid of such an approach are available. Examples of ILs applied are mono- (e.g., [C*_n_*C_1_im]Cl/Br, [C*_n_*C_4_im]Br and [C_7_H_7_C_1_im]Cl) and dicationic (e.g., [C_4_(C_n_im)_2_]Br_2_) imidazolium-based, together with [PF_6_]^–^ and [NTf_2_]^–^ salts as the ion exchange agent [152,153,154,155]. A hydrophobic IL containing the imidazolium cation and the [PF_6_]^–^ or [NTf_2_]^–^ anions from the parent IL and the ion exchange salt, respectively, is yielded, easily dispersing through the sample with no need of an extra dispersant [152,153,154,155]. As claimed with the extraction of tetracyclines from food samples [152], the analytical performance of in situ IL-DLLME using [C_7_H_7_C_1_im]Cl and NH_4_PF_6_ (CFs = 25–98 and limits of detection = 0.12–0.45 µg/L) matched or even overwhelmed that of other previously reported methods including conventional IL-DLLME using hydrophobic ILs and SPE. In the case of phenolic endocrine-disrupting chemicals in water samples, similar or higher CFs (140–989) were obtained by in situ IL-DLLME when compared with DLLME based on conventional organic solvents despite the lower limits of detection (0.8–4.8 ng/mL) [153].

In situ IL-DLLME can be hastened by the implementation of magnetic ILs, as previously discussed with IL-based ABS. Yao and Du [156] synthesized a series of imidazolium-based ILs containing a TEMPO functional group at the cation (i.e., [C*_n_*C_1_im-TEMPO]Cl, with *n* = 2, 3, 4, and 5). These were further combined with KPF_6_ as the ion exchange species to simultaneously determine five sulfonamides in milk samples. Upon the formation of the corresponding hydrophobic IL, a microdroplet enriched in the target analytes was quickly created, being further separated by the application of an external magnetic field, diluted, and directly injected in an HPLC apparatus bearing a UV detector. The proposed method was deemed precise, showing the high analytical performance (i.e., CFs in the order of 40, limits of detection ranging between 0.534 and 0.891 µg/L, and analytes recoveries higher than 95%) [156].

To sum up, applications of hydrophilic ILs in DLLME reveal a clear inclination to imidazolium-based ILs [148,149,150,151,152,153,154,155,156]. Thus, there is still room for further exploring other IL chemical structures, particularly as an attempt to overcome possible technical difficulties regarding practical execution and compatibility with the analytical equipment. If on the one hand hydrophilic ILs display a limited role as extraction solvents in IL-DLLME dealing with water samples (except for the in situ typology) [157], on the other hand, this aspect is particularly relevant within applications involving oil samples that remain a poorly explored approach using IL-DLLME [148,149]. In applications involving pharmaceuticals, if one considers the existence of oil-based pharmaceuticals and essential oils along with the need to analyze their pharmaceutical components, the search for ions bestowing ILs with better magnetic and viscosity features would contribute to significant achievements in the field. The use of hydrophilic ILs in DLLME as either dispersing agents or in in situ IL-DLLME allows one to reduce the amounts of volatile organic solvents in sample pretreatment techniques while respecting the principle of Green Analytical Chemistry [147]. However, the use of water-unstable [BF_4_]^–^ based ILs [158] as dispersing solvent in the DLLME of target analytes from aqueous samples is not recommended [150]. Moreover, using hydrophilic IL in in situ DLLME allows to outpace the high cost and the limited structural diversity of most common hydrophobic ILs, with structures of improved environmental and performance features [14,15,159]. It should be finally underlined that more than one pretreatment technique can be subsequently employed to meet the unmet requirements of a given application. In this domain, a noteworthy example comprises the application of IL-based ABS (extraction of the analytes) coupled with in situ IL-DLLME (purification of the analytes), where the ion exchange salt is added to the IL-rich phase of the ABS after extraction [160].

## 5. Recovery and Purification of Pharmaceuticals Using IL-Based Systems

**Figure 7:**
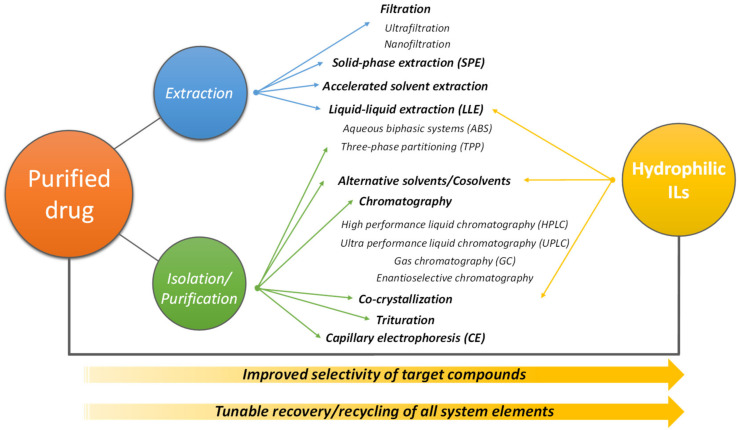
Schematic overview of the main strategies for developing drug extraction and purification approaches, highlighting the main applications of hydrophilic ILs as alternative solvents to overcome the main limitations associated with the current techniques.

VOCs have been commonly used in the pharmaceutical industry as reaction media, for the separation and purification of the manufactured products and the cleaning of equipment [161]. In the last years, many efforts have been placed aiming at reducing risks of exposure and release of hazardous materials, as well as through the replacement of non-hazardous materials [162]. ILs are one of the alternative classes of solvents (over VOCs, and particularly due to their non-volatile nature) that have been studied for the separation and purification of drugs [59,163,164,165,166]. Figure 7 summarizes the drug purification techniques available, which can be summed up to their general extraction for a specific medium [such as filtration, LLE, solid-phase extraction (SPE)] or their isolation from other components to a specific medium (such as alternative solvents, chromatography, co-crystallization). Based on the literature data, the works dealing with hydrophilic IL-based techniques for the extraction and separation of pharmaceuticals are distributed into three main approaches: (1) LLE mainly resorting to ABS, but also to conventional approaches using traditional organic solvents; (2) three-phase partitioning (TPP), and (3) co-crystallization processes, as represented in Figure 7. A survey of the information reviewed in the current section is given in Table 4, where pharmaceuticals recovered and purified using IL-based systems are listed.

### 5.1. IL-Based ABS

Albeit hydrophobic IL-water biphasic systems are the most studied drug separation techniques based on ILs as alternative solvents, various works addressing hydrophilic and more benign ILs have emerged in recent years [39,40,59,167]. As in the previous section, IL-based ABS and/or aqueous solutions of ILs have been investigated in the separation and purification of pharmaceuticals. IL-based ABS design should consider water-stable [4,8,12,168] and, whenever possible, more benign ILs [22,38,169,170], combined with various phase-forming agents, such as salts [28,120,126,171,172], polymers [39,172,173,174,175], surfactants [176], amino acids [177] and polar organic solvents [178,179]. Among these, Shahriari et al. [40] reported, for the first time, the existence of ABS composed of cholinium-based ILs and the inorganic salt K_3_PO_4_, followed by their application to separate tetracycline and its hydrochloride salt. Tetracycline preferentially partitions to the IL-rich phase; however, an opposite behavior was observed when using [N_111(2OH)_][Glt]. Although the partitioning behavior of antibiotics was explained by the high K_3_PO_4_ salting-out aptitude, the role of the IL structure could not be neglected in the case of [N_111(2OH)_][Glt]. On the other hand, Pereira et al. [39] employed PEG 600 and cholinium-based ILs to generate ABS for the pre-purification of tetracycline produced in a fermentation broth. Contrarily to the results reported by Shahriari et al. [40], Pereira et al. [39] demonstrated that antibiotics preferentially partition to the polymer-rich phase in most systems, thus highlighting the impact of the IL structure on the partitioning behavior of tetracycline. Nevertheless, the most significant issue of this work was the development of an alternative purification process of antibiotics from the production medium, i.e., a fermentation broth of *Streptomyces aureofaciens*, which revealed the potential of IL-based ABS as efficient pre-purification routes to be applied to real systems. The authors [39] emphasized the extraction of tetracycline to the polymer-rich phase as an economically viable pre-purification step: firstly, the components employed are relatively cheaper and biocompatible when compared to the use of other conventional structures of ILs; secondly, the selective partition of tetracycline to the polymer-rich phase would enable the removal of most part of the fermentation broth contaminants, in particular proteins, lipidic compounds, and other cell-debris. The authors further suggested the use of the polymer-rich phase, as the antibiotic extractive phase, to be further applied in chromatography techniques for polishing [39].

Commonly used inorganic salts present some drawbacks in the extraction of biomolecules that are susceptible to ionic strength. To provide milder extraction conditions, a seminal work [177] reported on the use of amino acids as alternative phase-forming agents of IL-based ABS. The role of IL-amino-acid-based ABS as extraction platforms was investigated by addressing the partition of caffeine, ciprofloxacin, and its hydrochloride salt. ABS composed of imidazolium-based ILs combined with proline or lysine were used, where the obtained partition behavior was mainly contingent on the IL anion structure despite the amino acid and target compound structure [177]. Although no real samples were studied, this work [177] contributed to enlarge the range of phase-forming agents available in the domain of IL-based ABS, particularly if the extraction of more labile biomolecules is envisaged.

With a different perspective, Silva et al. [180] recently published a new alternative process for the recovery of pharmaceutical ingredients from drug pills. For that, the authors transformed IL-based ABS into TPP systems composed of hydrophilic ILs and citrate-based salts to purify three NSAIDs, namely ibuprofen, naproxen, and ketoprofen. While NSAIDs preferentially partitioned to the IL-rich phase, the main bulk of the pill’s excipients was settled in the interface of the systems. After the extraction of the pharmaceutical ingredients, NSAIDs were isolated/precipitated by making use of anti-solvents, namely citric acid and aluminum sulfate aqueous solutions. Along with this study, the authors [180] also measured the relative stability of NSAIDs in ILs and ILs/salts mixtures towards the implementation of a new protocol adapted from the organization for economic cooperation and development (OECD) guidelines. The authors [180] found no significant loss of NSAIDs stability during the five days of experiments. Despite there is no further experimental work regarding the recycling and the use of ABS components, IL-based TPP are envisaged as sustainable and efficient alternatives for the recovery of a plethora of APIs from pharmaceutical wastes. Accordingly, the same group [167] published a pioneering work where ABS composed of ILs were applied for the extraction of paracetamol, which presence has been highly targeted within pharmaceutical wastes. In this work, novel ABS composed of tetraalkylammonium-based ILs and three salts, viz., C_6_H_5_K_3_O_7_/C_6_H_8_O_7_ and K_2_HPO_4_/KH_2_PO_4_ buffers at pH ≈ 7 and K_2_CO_3_, were investigated. Optimization studies, comprising the ammonium-based IL chemical structure, salt structure and concentration, and TL length and pH, were firstly carried out with the pure compound. Under this scenario, the best conditions were then used to extract paracetamol from expired pills (considered as standard materials in several industries), where complete extraction efficiencies of the API were achieved using ABS composed of [N_2222_]Br or [N_4444_]Br + K_2_CO_3_ or C_6_H_5_K_3_O_7_/C_6_H_8_O_7_ at pH ≈ 7. A different strategy to valorize unused and outdated medicines was recently proposed by Dimitrijević and coworkers [175], who took full advantage of the distinct capacity of polymer-salt- and polymer-IL-based ABS to extract active pharmaceutical ingredients (viz., acetaminophen, caffeine, and theophylline). Polymer-C_6_H_5_Na_3_O_7_-based systems showed a higher extraction aptitude, being firstly used to recover all three compounds, whereas the polymer-[C_4_C_1_im]Cl-based system was used for fractionation purposes. Following a solid-liquid extraction step using a polymer aqueous solution to extract the target pharmaceuticals from their solid formulations, all three pharmaceutical compounds were recovered (>79%) using polymer-C_6_H_5_Na_3_O_7_-based systems. The polymer-rich phase enriched in the target products was further used to prepare the polymer-[C_4_C_1_im]Cl-based system aiming the separation of caffeine and acetaminophen present in the same pharmaceutical formulation. Although no complete separation was achieved (recovery efficiencies of 70.49% and 65.70% for acetaminophen and caffeine, respectively), the proposed integrated process allowed to remove excipients present in the pills [175]. It should be finally underlined that the authors used an amphiphilic copolymer belonging to the Pluronics^®^ family (Pluronic PE 6200), which bears ethylene oxide (hydrophilic) and propylene oxide (hydrophobic) units organized in a triblock structure. By altering the relative number of each type of units and the molecular weight, copolymers can be finely tuned to afford intermediary or even new physicochemical properties as compared to their corresponding homopolymers (i.e., PEG and PPG), thus allowing the design of efficient and selective ABS.

IL-based ABS have been also proposed by Almeida et al. [163] to remove pharmaceutical contaminants from wastewaters, namely diclofenac, ibuprofen, naproxen, and ketoprofen. Although with a different perspective of the previously published works regarding the use of IL-based ABS for the extraction and purification of pharmaceuticals, the authors proposed an integrated and highly efficient ABS-based strategy to remove persistent pollutants from an aqueous environment in a final stage of a WWTP. Al_2_(SO_4_)_3_ was used as the salting-out agent in IL-based ABS as WWTPs currently use this salt for the purification of drinking water, namely as a flocculating agent. After an optimization step regarding the effect of the IL chemical structure, extraction efficiencies of NSAIDs up to 100% into the IL-rich phase in a single-step for the [P_4441_][C_1_SO_4_]-rich phase have been achieved. The ABS composed of [P_4441_][C_1_SO_4_] + Al_2_(SO_4_)_3_ was therefore selected to investigate drug recovery and IL reusability steps. The recovery of NSAIDs was designed based on the ability of ILs to act as hydrotropes, i.e., to increase the water solubility of hydrophobic drugs in several orders of magnitude (from 300- to 4100-fold when compared with pure water for the studied pharmaceuticals). In this context, the saturation of the IL-rich phase with each pharmaceutical was reached, followed by induced precipitation of the drug by the addition of water that acts as an anti-solvent. This approach allowed to recover up to 91% of the dissolved drugs, and the IL recovery and reuse (with >94% of the IL recovered and reused in four consecutive cycles). By working at the compositions employed, the authors [163] estimated the possibility to treat 3319 L of water with 1 g of [P_4441_][C_1_SO_4_] (having into account the saturation values of the drugs in the IL-rich phase). Nevertheless, it should be stressed that the authors [163] did not apply the proposed technology to real wastewater samples, which is essential for proving the feasibility of the developed process for real matrices. After the “cleaning” of the IL-rich phase by the induced precipitation of pharmaceuticals, the phase-forming components were recovered and reused in four consecutive cycles. NSAIDs precipitation and IL recovery and reuse are of high relevance towards their application at an industrial level. This situation is described in more detail in a recent review [13], where the authors hypothesized integrative processes for most relevant works, comprising the production, separation/purification, and isolation of drugs and reusability of the phase-forming components in IL-based ABS. However, a significant lack of information persists in the literature regarding the potential scale-up of the developed technologies. With a similar goal of removing contaminants from effluents, Álvarez et al. [176] used ABS composed of [N_111(2OH)_]Cl and non-ionic surfactants (i.e., Tween 20 and Tween 80) to remove two NSAIDs (viz., ibuprofen and diclofenac) from aqueous solutions. Following the establishment of liquid-liquid regimes through the ABS phase diagrams determination and characterization at different temperatures, the most hydrophobic surfactant, i.e., Tween 80, was selected to perform NSAIDs extraction studies. Regardless of the temperature and biphasic mixture composition, extraction efficiencies higher than 90% towards the surfactant-rich top phase were obtained for both NSAIDs, especially ibuprofen due to its higher lipophilic character. Again, no studies involving real environmental samples were done by the authors; still, a hypothetical process diagram was proposed envisioning the treatment of effluents arising from soil wash with a Tween 80 aqueous solution [176]. It is important to highlight that the choice of the phase-forming agents should be performed to better integrate the intended application, as done with Tween, which is used in contaminated soil remediation processes [181] and Al_2_(SO_4_)_3_ that assists water purification processes [163].

Even though a full characterization of the solid compounds obtained after the NSAIDs precipitation step was not verified in the aforementioned works [167,180], the crystalline structure and polymorphs recovered from the IL-rich phase require special attention since these can lead to variations in the drug performance, such as reduction of solubility/bioavailability and dissolution rates [182]. In this context, crystallization must be strictly controlled if the goal is to reach the pharmaceutical industry, which was not the case of the previously described works.

Further expanding the diversity of phase-forming components of IL-based ABS and pharmaceuticals recovered, the use of polar organic solvents as well as antidepressants and hormones was recently reported by Lima’s research group [178,179]. A series of ILs belonging to the imidazolium family, while bearing distinct anions and cation alkyl chain lengths ([C*_n_*C_1_im]X, with *n* = 2–10 and X = Cl^–^ as well as *n* = 2 and X = [C_1_CO_2_]^–^, [(CH_3_O)_2_PO_2_]^–^, [C_1_SO_3_]^–^, Cl^–^, Br^–^, [TsO]^–^, [SCN]^–^, and [N(CN)_2_]^–^) was studied regarding their aptitude to form ABS with 1,3-dioxolane, 1,4-dioxolane, acetone and acetonitrile [178]. While ILs with higher hydrophobic nature ([C_2_C_1_im]X, with X = [TsO]^–^, [SCN]^–^, and [N(CN)_2_]^–^) failed to form ABS with 1,3-dioxolane, the most hydrophilic [C_2_C_1_im][C_1_CO_2_] studied displayed the best ability. Excepting those unable to afford a liquid-liquid system, all remaining [C_2_C_1_im]-based ILs were used to extract three antidepressants, namely fluoxetine hydrochloride, sertraline hydrochloride, and paroxetine hydrochloride from aqueous solutions. Both the IL and antidepressant under investigation played a role in the partition behavior. In specific systems, it was possible to delineate strategies to separate pairs of antidepressants based on their selectivity towards each phase. For instance, the most selective ABS composed of [C_2_C_1_im][C_1_CO_2_] and 1,3-dioxolane affords a selectivity of 5.83 in the separation of paroxetine hydrochloride and fluoxetine hydrochloride [178]. On the other hand, using protic ILs and acetonitrile, authors [179] focused on the extraction of four female hormones (17 β-estradiol, estriol, EE2, and progesterone) from aqueous solutions. The binodal curves of systems composed of two protic ILs ([N_000(2OH)_][C_1_CO_2_] + [N_00(2OH)(2OH)_][C_1_CO_2_] and [N_000(2OH)_][C_1_CO_2_] + [N_00(2OH)(2OH)_][C_3_CO_2_]) at distinct compositions (0–100%), acetonitrile and water were determined. The aptitude of ABS formation was again dictated by the hydrophilicity of the IL, i.e., the introduction of hydroxyethyl group at the protic ammonium cation and the elongation of the anion alkyl chain seemed to limit the ABS formation. By comparing the performance of only one protic IL and their mixtures as extraction platforms, the latter was deemed superior with maximum extraction efficiencies (over 99.9% for all hormones). Overall, the extraction efficiencies and selectivity were contingent on the protic ILs structure, protic IL mixture composition, and the target hormone, with progesterone and 17β-estradiol migrating to the acetonitrile-rich top phase, and the remaining hormones partitioning towards the protic IL-rich phase. The recovery and reuse of the ABS phase-forming agents remain a challenge in this domain despite the attempt of the authors [179] to suggest a possible route to be followed. Accordingly [179], the acetonitrile-rich phase could be recovered by pervaporation, while precipitation with water followed by pervaporation would enable the IL-rich phase and the antisolvent recovery and reuse. Still, it should be noted that authors [179] reported a partition coefficient of the protic ILs close to 1, meaning that these are evenly distributed throughout the system and may pose additional challenges during recovery and reuse operations.

### 5.2. Other Techniques

Despite the intensive research using hydrophilic ILs and ABS in fields involving the recovery and purification of pharmaceuticals, other techniques are worthy to be mentioned. As aforementioned, hydrophilic ILs can also be applied in the development of LLE techniques using IL-immiscible organic solvents and in crystallization approaches.

One of the main challenges in the continuous manufacturing of pharmaceuticals is the need to obtain the final product free of contaminants (e.g., starting precursors, intermediates, solvents) and to recycle expensive unreacted starting materials. The solvency power of ILs towards a multitude of compounds led Rogers and co-workers [183] to develop a separation process for an intermediate of the aliskiren synthesis from its main contaminants. The main objective of the authors [183] was to overcome the technical complexity, the difficulty of being implemented in continuous manufacturing, and the limited performance of common separation processes. Authors [183] tested LLE systems composed of the hydrophilic IL [C_2_C_1_im][C_1_CO_2_] with ethyl acetate or *n*-heptane versus those bearing the hydrophobic IL [C_2_C_1_im][NTf_2_] with *n*-heptane or water. Limited purity of the intermediate and expensive starting material was obtained with the [C_2_C_1_im][NTf_2_] + water system due to contamination with IL. By using the hydrophilic IL [C_2_C_1_im][C_1_CO_2_] with ethyl acetate, purity limitations were surpassed since the hydrophilic IL was more easily removed after washing with water. It should be mentioned that when moving from standard to the real reaction mixture, new technical challenges emerged. Due to the existence of byproducts in the mixture, an extra washing step with *n*-heptane was introduced [183].

According to the literature, the crystallization of pharmaceuticals can be divided into two main approaches: precipitation with anti-solvents and cooling crystallization, with several works addressing crystallization strategies conducted in IL media based on these two approaches. Kroon et al. [184] work opened the way to test the conditions of crystallization of methyl-(Z)-α-acetamido cinnamate in IL media using supercritical CO_2_ as anti-solvent, by lowering the solubility of N-acetyl-(S)-phenylalanine methyl ester in [C_4_C_1_im][BF_4_]. This product results from the asymmetric hydrogenation of methyl-(Z)-α-acetamido cinnamate, an intermediate in the production of Levodopa, a drug used in Parkinson’s disease. In a first attempt, the authors determined the phase behavior of the ternary system composed of [C_4_C_1_im][BF_4_], CO_2_, and methyl-(Z)-α-acetamido cinnamate. It was concluded that CO_2_ can act as either a co-solvent or anti-solvent in distinct concentrations, depending on if the system performs at low concentrations of CO_2_ (30 mol%) or at high CO_2_ concentrations (40–50 mol%), respectively. In another work, Weber et al. [185] focused on the purification of paracetamol by its dissolution using ILs and further crystallization induced by anti-solvents. To improve the crystallization process, the manipulation of the hydrogen bonding interactions for tailoring the solubility of paracetamol and its main impurities (4-aminophenol, 4-nitrophenol, and 4′-chloroacetanilide) was firstly performed in IL media. In particular, ILs composed of anions of increasing hydrogen bond basicity ([NTf_2_]^–^, [BF_4_]^–^ and [C_1_CO_2_]^–^) and cations with increased hydrogen bond acidity ([C_4_pyr]^+^, [C_4_C_1_im]^+^, [C_2_C_1_im]^+^ and [OHC_2_C_1_im]^+^) were tested, where it was found that the hydrogen bond basicity of the anion plays a dominant role in the crystallization of paracetamol. [C_2_C_1_im][C_1_CO_2_] showed the best ability to solubilize paracetamol. To understand the molecular interactions occurring in IL media for the proper design of crystallization processes, the authors [185] then investigated mixtures formed by [C_2_C_1_im][C_1_CO_2_] and a less viscous IL, namely [C_2_C_1_im][NTf_2_]. The authors found out that the [C_2_C_1_im][C_1_CO_2_]*_x_*[NTf_2_]_1–*x*_ ability to solubilize paracetamol and 4-aminophenol linearly correlates with the [C_1_CO_2_]-based IL concentration. Furthermore, spectroscopic studies demonstrated the role of hydrogen bonding in the described phenomenon. Finally, three strong hydrogen bond donating compounds were studied as anti-solvents, namely ethanol, acetic acid, and 1,1,1,3,3,3-hexafluoroisopropanol. It was found that with the proper choice of the anti-solvent, the co-precipitation of impurities associated to the target drug can be controlled.

Despite Almeida et al. [163] followed the use of the potentiality of ILs as hydrotropes to precipitate NSAIDs from more complex aqueous systems, particularly IL-based ABS, only in a very recent work, the same research group evaluated the impact of the IL medium on the crystallization of ibuprofen [59]. For that purpose, suitable crystals of ibuprofen precipitated in ethanol and [C_4_C_1_im][SCN] were analyzed by powder and single- crystal X-ray diffraction and optical microscopy. The unit cell agrees with its crystallographic data reported in Cambridge Crystallographic Data Centre (www.ccdc.cam.ac.uk/data_request/cif, accessed on 21 April 2021) and the authors observed no significant differences in the power diffractogram. Nevertheless, it should be noted that ibuprofen crystals precipitated from both solvents demonstrated different crystal habits from ibuprofen crystal morphology [186,187]. When the crystallization was taken in ethanol medium, plate-shaped, and some tube-shaped crystals were observed, while crystals formed in the IL medium showed needle- and plate-shaped morphologies. One important aspect is the need for high-throughput technologies able to predict and characterize crystal structures, namely crystal size distribution, crystal shape, and polymorphic forms produced [182]. Moreover, differences in lab-scale and industrial-scale crystallization pose scale-up challenges, in areas such as hydrodynamics, and heat and mass transfer performance. Scaling-up crystallization strategies should be adopted in research areas since significant changes in nucleation, growth, breakage, and agglomeration affect the crystal quality. In conclusion, several opportunities and challenges exist in the design of isolation strategies; for instance, crystallization could be integrated with the techniques described in previous sections, which will help researchers to achieve a more precise control process, constant drug final product quality, and efficient process operation.

## 6. Key Factors in Choosing Hydrophilic ILs in Fields Involving Pharmaceuticals

Despite all the advances made hitherto by using ILs, it became clear that unmet needs in pharmaceutical applications are only achieved if ILs are properly designed. By playing with their constituent ions, ILs are bestowed with adequate physical, chemical, and biological features fitting the operating requirements and performance of the intended application with eco-, bio-, and cost-friendlier credentials. Therefore, the choice of the ILs should ponder on a wide range of factors, including properties, technical specificities, environmental impact, costs, among others. To help guide decision-making in the development of applications involving pharmaceuticals, particularly within the four domains here overviewed, brief notions on key factors affecting the choice of the IL are provided in this section.

### 6.1. Cytotoxicity

An important factor to be considered is the cytotoxicity of ILs that is essential to validate their potential to be applied as drug solubility enhancers and as carrier agents in drug delivery devices. Several studies on cytotoxicity of ILs towards various cell lines are nowadays available, e.g., with human keratinocyte cell line HaCaT [105], colorectal adenocarcinoma cell lines CaCo-2 [188,189] and HT29 [29,190], and human cervical carcinoma cell line HeLa [191,192]. The accumulated IL cytotoxicity data, ranging from micromolar (or even nanomolar) to millimolar scales, suggest the main dependence on their chemical structure despite the important role played by the cell line (for recent reviews on the topic refer to [14,193,194]). Structural modifications at the level of the ILs’ ions can be somehow correlated with their cytotoxicity, allowing to establish general guidelines on how to design more biocompatible ILs.

Longer alkyl side chain lengths (i.e., more hydrophobic ILs) yield more cytotoxic ILs as verified with [C*_n_*C_1_im]Br, [C*_n_*C_1_im]Cl, [N_11*n*(2OH)_]Br, [N_222*n*_]Br and [C*_n_*Car]Br over distinct cell lines [HeLa, rat pheochromocytoma (PC12), CaCo-2, HaCaT] [71,195,196,197]. The introduction of functional groups, namely oxygenated moieties, leads to less cytotoxic ILs as shown with imidazolium-based ILs bearing different anions against rat promyelocytic leukemia (IPC-81) and PC12 cell lines [198,199]. More lipophilic and fluorinated anions are more prone to produce cytotoxic IL than non-fluorinated anions (e.g., Cl^–^), as demonstrated with imidazolium-based ILs towards the IPC-81, CaCo-2, and the murine fibroblast (NIH/3T3) cell lines [189,200]. Regarding the cation core role, cholinium-based ILs generally embrace low cytotoxicity, even when compared to other ILs, such as imidazolium-, pyridinium- and phosphonium-based ILs [38,99,170,201]. This explains the recent interest given to this type of ILs in drug solubility enhancement and drug delivery applications [64,70,99]. Despite the higher cytotoxicity of [P_666,14_][Ger] and [P_666,14_][C_5_CO_2_] against the normal human bronchial epithelial (NHBE) cell line than their cholinium-based counterparts, Zakrewsky et al. [99] found the opposite behavior with the [Ole]^–^ anion when developing a transdermal drug delivery system. Along with cholinium-based ILs, other types of naturally-derived ILs, such as those based on (or derived from) amino acids, have gained momentum as more biocompatible options. ILs composed of the [N_111(2OH)_]^+^ cation and amino-acid-based anions were shown to be less cytotoxic against the HeLa cell line than their imidazolium-based congeners [201]. Yet, some ILs bearing amino-acid-derived anions or cations led to “an unexpected increase of toxicity” over CaCo-2 and the murine fibroblast (NIH/3T3) cell lines when compared to common imidazolium-based ILs [189]. The picture emerging from these results suggests that general guidelines should be used with caution, always considering plausible synergistic effects of the cation-anion pair and the tested cell line or organism. Moreover, generalities considering the safer character of ILs should be avoided and the selection of ILs should take into account tangible information.

It should be additionally mentioned that cholinium-based ILs have been recently reported as a promising class of biocompatible ILs to enhance the stability of biopharmaceuticals, namely immunoglobulins and other proteins [22,32] and nucleic acids [23,35,202], only depending on their proper design. Together with their cytotoxic features, this scenario pinpoints the potentiality of this type of ILs in biopharmaceutical formulations, as shown with [N_111(2OH)_][Ger] and insulin [203].

The selection of ILs for drug delivery systems may be difficult if the structure fitting the technical requirements tends to pose cytotoxic effects. To reach a compromise between the IL technical performance and biocompatibility, the application of quantitative structure-activity relationship (QSAR) models may be useful [204]. If on the one hand, the diversity of ILs available makes them more prone to be finely tuned for a specific application, on the other hand, trial-and-error assays may be needed to find the most adequate structure; by using QSAR models, the process of optimizing the IL structure may be expedited. Another useful route to aid the design of more biocompatible ILs is by unveiling their mechanisms of action towards living cells (for a recent review on the topic refer to [194]).

In general, it should be pointed out that “toxic” and “non-toxic” terms must be carefully employed when dealing with ILs for drug delivery applications. In fact, there is a current lack of information regarding in vivo studies involving ILs [205], while significant discrepancies still exist for different test cells when carrying out in vitro assays regarding the same IL structure.

### 6.2. Environmental Risks

During a more initial stage of the ILs research, these solvents aroused interest as VOCs substitutes due to their negligible volatility, low flammability, and improved thermal stability [2]. Consequently, ILs started to be deemed as “green solvents” and used to renew chemical- and pharmaceutical-related methods, processes, and products in the light of Green Chemistry and Green Analytical Chemistry [1,147]. These features together with their high solvation aptitude for a plethora of molecules placed ILs as useful tools for manufacturing processes in the pharmaceutical industry (e.g., in recovery and purification processes) and sample preparation techniques. The solubility in water of most ILs (at least to some degree), especially that of hydrophilic ILs, together with their toxicological features, highlighted their potential to enter the environment and negatively affect ecosystems [14,193]. Under this scenario, ILs are currently classified as emerging contaminants like other well-known pollutants, such as perfluorinated alkyl compounds [206]. In addition, the presence of (imidazolium-based) ILs in the environment is a reality with potential health hazards, as shown with in vivo tests with mammals [207,208].

Based on the information provided, the design of ILs with eco-friendlier credentials remains critical. The main guidelines previously provided to develop less cytotoxic ILs also apply here to some extent. Ecotoxicity studies involving ILs are mainly performed with organisms of different taxonomic groups, namely bacteria (e.g., *Aliivibrio fischeri*), producers (e.g., *Raphidocelis subcapitata* and *Lemna minor*), primary consumers (e.g., *Daphnia magna*), and secondary consumers (e.g., *Danio rerio*) [209,210]. Generally, the hydrophobicity of the cation is the main factor influencing ecotoxicity, with shorter alkyl side chains, functionalization with polar substituent groups, and water-soluble cations (yet, non-aromatic) being at the forefront of “benign by design” strategies [14,193]. Despite being less preponderant, the role of the anion upon the ecotoxicity can be reduced by prioritizing halides or organic acids instead of fluorinated anions [14,193].

In addition to toxicity, the biodegradability of ILs is an important parameter to understand environmental impacts and fate. Among structural features contributing to an easier biodegradation of ILs are relatively long alkyl chains and oxygenated units; however, it should be mentioned that as with toxicity, the test organism and the synergy between the cation and the anion should not be ignored [14,193]. Even so, most common ILs, including [C*_n_*C_1_im]Cl-based ILs, are not readily biodegradable, reinforcing the need for alternative ILs [14,193]. However, the ways by which the alkyl chain length allows designing less toxic and more biodegradable ILs somehow work oppositely [14,193], being necessary to make a trade-off between both. To this aim, QSAR models may also be useful.

The stability of ILs is also important from an environmental fate and pathways perspective. In this domain, it is important to call the attention to the moderate to high propensity of the [PF_6_]^–^ and [BF_4_]^–^ anions in imidazolium-based ILs to be hydrolyzed [158]. While a few authors [58,137,138,139] have used such ILs in applications involving water, their lack of stability in aqueous milieu may restrict their implementation in ABS and/or as hydrotropes/cosolvents.

Due to the low biodegradability and the increasingly frequent use of ILs as solvents in both academia and industry, the importance of treating wastewater streams containing ILs (upon disposal) becomes evident. To tackle this issue, several wastewater treatment technologies are available, entailing the removal of the IL by adsorption with activated carbon, membranes, and IL-based ABS as well as its degradation by chemical-, photochemical-, electrochemical- and thermal-based methods [211].

To foster the implementation of ILs in pharmaceutical applications, several stages of ILs application, i.e., from their preparation until being released, should be appraised by using life cycle assessment tools [212]. Other green metrics can be additionally employed, such as the E-factor, which profiles the amount of waste generated per amount of product manufactured [213]. Both fine chemicals and pharmaceuticals industrial sectors have high E-factors (i.e., 5 to 50 and 25 to >100, respectively [213]), which may be solved if ILs are applied, for instance, in recovery and purification processes [214]. In the case of IL-assisted pharmaceuticals analysis, the environmental and health impacts can be determined by the HPLC-EAT (Environmental Assessment Tool), which considers all the solvents used in the chromatographic method, including whenever possible the sample pretreatment step [215].

Finally, to be underlined is the fact that the well-documented [C_2_C_1_im][C_1_CO_2_] is a substance registered in the Registration, Evaluation, Authorization, and Restriction of Chemicals (REACH) [216]. This achievement represents a steppingstone to the implementation of ILs within industrial and commercial settings.

### 6.3. IL Recovery and Reuse

In the domain of pharmaceutical compounds, the recovery and reuse of the IL have a positive impact on both environmental and economic features of IL-based processes. This aspect is particularly relevant within recovery and purification processes, where larger amounts of ILs and their aqueous solutions are used if compared to the remaining fields of application here discussed. In the works here discussed, particularly those focused on ABS, isolation routes for the target NSAIDs were developed and optimized, mainly resorting to precipitation with antisolvents [163,180]. After the isolation step, the recovery of the IL or the IL-rich phase and further reuse was carried out with success at least throughout 4 cycles [163]. In addition to precipitation with antisolvent, there are other IL recovery technologies available. Depending on the type of technique under investigation (IL-based ABS or other) and the application envisaged, distillation, back-extraction, sorption-based techniques, and application of external stimuli can be adopted [217]. In the domain of external stimuli, IL recovery can be easily achieved by taking full advantage of magnetic ILs, as described with sample pretreatment techniques [145,146].

### 6.4. Cost

Despite being possible to overcome the costly character of some ILs by employing recovery and reuse strategies, the cost of the IL remains a challenge. In this sense, ammonium-based ILs, which are less expensive than imidazolium-based ones, while providing less (cyto)toxic solvents, have started to mark their own way [14,15]. The formulation of cheaper ILs should also consider the anion moiety, where halides (Cl^–^ and Br^–^) as well as [C_1_CO_2_] ^–^ and [C_1_SO_4_]^–^ should be the first choices [15]. However, the narrow structure availability of more economically appealing IL cations, mainly ammonium- and phosphonium-dependent, raises an urgent need for finding/synthesizing novel structures vis-à-vis the higher structural diversity of natural-based anions available. In this domain, natural-based anions are expected to become cheaper IL constituents.

Besides the cost of the IL itself, the economic evaluation of the IL-based processes developed is paramount to disclose any economic vulnerability prior to industrial implementation. In this framework, Torres-Acosta et al. [218] performed an economic analysis for the primary recovery of tetracycline in batch mode employing different ABS. Results obtained demonstrated the improvements in using IL-based ABS when compared to more traditional ABS from an industrial point of view, although additional research, namely on extending the use of IL-based ABS, is still needed to further improve the tetracycline purification and IL removal [218].

## 7. Conclusions and Future Prospects

In the past years, ILs have raised high interest in academic research and industry in several areas, including the pharmaceutical field. In this field, the use of hydrophilic ILs and their aqueous solutions instead of hydrophobic ones is a preferred choice. In this work, we have reviewed and discussed the main results of relevant works mainly published in the last decade within pharmaceutical-based applications, namely: (i) improvement of pharmaceuticals solubility, envisioning improved bioavailability; (ii) IL-based drug delivery systems; (iii) pretreatment strategies of samples comprising pharmaceuticals to improve analytical methods performance; and (iv) recovery and purification of pharmaceuticals using IL-based systems.

As discussed above, many works have highlighted that ILs are efficient solvents for a myriad of pharmaceutical applications. ILs based on imidazolium cations remain the most reported, even if more recently quaternary ammonium-based ILs (mainly, cholinium-based) of improved eco- and bio-friendly nature started to gain momentum. Cholinium-based ILs as well as other bioinspired structures hold great potential in fields involving pharmaceuticals. The performance of cholinium- and carnitine-based ILs, if properly designed and the process optimized, is highly promising. In the improvement of pharmaceuticals solubility, these ILs were able to increase the water solubility of poorly soluble compounds in at least two orders of magnitude, outpacing conventional salt hydrotropes and surfactants [70,71]. In the development of drug delivery systems, a low cytotoxic cholinium-based IL was used to produce topical and transdermal formulations containing antibiotics with success in terms of drug delivery efficiency as well as wound healing causing no skin irritation [99]. In the recovery and purification of pharmaceuticals, protic ammonium- and quaternary ammonium-based ILs have shown high performance and selectivity, allowing the development of more cost-effective processes (e.g., [218]). In turn, in the field of sample pretreatment and preconcentration approaches using IL-based ABS, as the amounts of ILs used are comparatively small, the opportunities of using this kind of ILs could be mainly justified by technical specificities, preconcentration performance, or compatibility with analytical equipment; still, in the domain of IL-DLLME, the diversification of the studied ILs could be valuable.

In applications involving IL-based ABS, there is a lack of studies regarding the use of more complex and real matrices: if on the one hand preconcentration studies are mainly focused on real samples (e.g., environmental waters, human fluids, food), even if spiked, on the other hand, recovery and purification related works mainly focus on standard solutions. In addition, and although concentration steps using IL-based ABS can offer more efficient pretreatment routes in analytical assays for environmental monitoring or diagnosis/prognosis due to the low amounts of samples used, the same does not apply when envisaging extraction and purification routes of APIs using ABS, where a significant lack of information persists in the literature regarding these systems scale-up. In this field, technoeconomic analysis and life cycle assessment remain in high demand. The integration of drug crystallization processes with the remaining (scale-up) steps upon the development of IL-based techniques would be a must approach to gather a deeper understanding of the crystal morphology of pharmaceutical drugs, which will certainly help researchers to achieve a more precise control process, constant drug final product quality and efficient process operation.

The use of ILs in the pharmaceutical field opens the door to advanced processes based on their tailor-made nature, which lights up one important question: how does the task-specificity allied to ILs will allow the expansion from the bench to an industrial view? Although the number of published scientific works and patents regarding ILs with potential use within the pharmaceutical field has significantly increased over the last years, the market size of ILs in the pharmaceutical industry is still almost nonexistent. According to an industry analysis report on ILs market size by application published in the Global Market Insights [219], in 2014, only 3.6% of the ILs market comprises pharmaceutical applications, representing the smallest piece of this market size. The ILs entry in pharmaceutical manufacturing processes has been conducted at a slow rate and several reasons might be hampering their application, from the early steps of drug manufacturing (namely, extraction and purification) to the most advanced steps of clinical trials. Although hydrophilic ILs have started to replace many applications of hydrophobic ILs due to their lower “toxicity” and “higher tunability” as well as their ability to set up biological-based microenvironments capable to fit within the “therapeutic” activity of the pharmaceutical ingredient, this review reflects that many of the challenges remain at bench-scale, and they must be primarily overcome before going through a large-scale overview.

Future research lines on IL-based processes within pharmaceutical applications must be aware of the previously discussed topics and bring up stakeholders to the scenario to raise attention to real problems outside academia. Process integration, scale-up, cost-effectiveness, biocompatibility, and low (cyto)toxicity, as well as the recovery and reusability of ILs, are mandatory aspects to bring effective improvements in comparison to current commercial technologies.

## Figures and Tables

**Table 1:** Application of hydrophilic ILs to improve the solubility (bioavailability) of pharmaceuticals.

Pharmaceutical/Compound	Hydrophilic IL	Solubility in Water (mg/L)	Maximum Solubility in IL Aqueous Solution (mg/L)	Operating Conditions		Ref.
				T (K)	*w*_IL_ *^a^*	
Albendazole	[C_4_C_1_im][BF_4_]	5.31 × 10^−1^	3.10 × 10^2^	298	0.33	[58]
	[C_6_C_1_im][BF_4_]		1.26 × 10^3^			
	[C_6_C_1_im]Cl		2.11 × 10^3^			
Vanillin	[C_4_C_1_im][TsO]	1.11 × 10^4^	4.46 × 10^5^	303	0.5	[17]
	[C_4_C_1_im]Cl		3.75 × 10^5^		0.8	
	[C_2_C_1_im][N(CN)_2_]		3.95 × 10^5^		0.5	
	[C_4_C_1_im]Cl		1.34 × 10^5^	298	0.3	[71]
	[C_4_Car]Br		6.09 × 10^4^		0.23	
	[C_6_Car]Br		9.43 × 10^4^		0.25	
Gallic acid	[C_4_C_1_im]Cl	1.44 × 10^4^	2.88 × 10^5^	303	0.5	[17]
	[C_4_C_1_im][N(CN)_2_]		3.30 × 10^5^		0.5	
Ibuprofen	[C_4_C_1_im][N(CN)_2_]	3.76 × 10^1^	4.47 × 10^3^	303	0.2	[59]
	[C_4_C_1_im][TsO]		2.01 × 10^3^		0.3	
	[C_4_C_1_im][SCN]		2.29 × 10^3^		0.2	
	[N_111(2OH)_][Van]		1.88 × 10^4^		0.8	[70]
	[N_111(2OH)_][Gal]		1.71 × 10^4^		0.65	
	[N_111(2OH)_][Sal]		2.26 × 10^5^		0.78	
Artemisinin	[C_4_C_1_im][N(CN)_2_]	6.18 × 10^1^	2.85 × 10^4^	303	0.9	[61]
	[C_4_C_1_im][TsO]		4.14 × 10^3^		0.6	
	[C_4_C_1_im][SCN]		1.91 × 10^4^		0.8	
Amphotericin B	[C_6_NH_3_][C_1_CO_2_]	2.00 × 10^−1^ [72]	2.50 × 10^2^	298	N.R.*^b^*	[50]
Itraconazole	[m-PEG_350_-NH_3_][C_5_CO_2_]	1.00 × 10^−6^ [73]	1.00 × 10^2^		N.R.*^b^*	
Glibenclamide	[N_111(2OH)_][Trp]	(1.50–2.40) × 10^1^ [74,75,76]	9.89×10^3^	310	0.07	[64]
Lamotrigine	[C_6_C_1_im]Br	1.70 × 10^2^ [77]	5.51 × 10^8^	313	0.8	[62]
Acetaminophen	[C_6_C_1_im]Br	2.49 × 10^4^	8.91 × 10^4^	313	0.15	[63]
Naproxen	[N_111(2OH)_][Van]	3.19 × 10^1^ [60]	1.78 × 10^4^	303	0.6	[70]
	[N_111(2OH)_][Gal]		2.00 × 10^4^		0.6	
	[N_111(2OH)_][Sal]		1.35 × 10^3^		0.78	

*^a^w*_IL_—IL weight fraction; *^b^* N.R.*—*Not reported.

**Table 2:** Application of hydrophilic ILs in the development of drug delivery systems.

Pharmaceutical/Compound	Hydrophilic IL	Drug Delivery Strategy	Ref.
Acyclovir	[C_1_C_1_im][(CH_3_O)_2_PO_2_]	(Tween-80 + Span-20) + IL microemulsions	[104]
	[C_1_C_1_im][(CH_3_O)_2_PO_2_]; [C_2_C_1_im][BF_4_]	(Tween-80 + Span-20) + IL microemulsions	[47]
Methotrexate	[C_1_C_1_im][(CH_3_O)_2_PO_2_]; [C_2_C_1_im][BF_4_]	(Tween-80 + Span-20) + IL microemulsions	[47]
Dantrolene sodium	[C_1_C_1_im][(CH_3_O)_2_PO_2_]; [C_2_C_1_im][BF_4_]	(Tween-80 + Span-20) + IL microemulsions	[47]
Lidocaine hydrochloride	[C_12_C_1_im]Cl; [C_14_C_1_im]Cl	micellar-based systems	[114]
Rutaecarpine	[C_12_C_1_im]Br	shaped iongels	[81]
Ibuprofen	[C_4_C_1_im][Ibu]	silica-based iongels	[96]
5-Fluorouracil	Cholinium Polyacrylate crosslinked structures	stimuli-responsive nanogels	[95]
Mannitol	[N_111(2OH)_][Ger]	Transdermal drug delivery	[99]
Cefadroxil	[N_111(2OH)_][Ger]	Transdermal drug delivery	[99]
Ceftazidime	[N_111(2OH)_][Ger]	Topical formulation	[99]

**Table 3:** Application of hydrophilic ILs in the pretreatment/concentration of pharmaceuticals to improve analytical analysis.

Pharmaceutical/Compound	Hydrophilic ILs	Sample to Be Analyzed	CF (-Fold)	System Components	Ref.
IL-based ABS					
Testosterone	[C_4_C_1_im]Cl	human urine	10	IL + K_2_HPO_4_	[132]
Epitestosterone	[C_4_C_1_im]Cl	human urine	10	IL + K_2_HPO_4_	[132]
Serum albumin	[C_4_C_1_im]Cl	human urine	20	IL + K_2_HPO_4_	[133]
BPA	[N_111(2OH)_]Cl	synthetic urine	100	IL + K_3_PO_4_	[129]
	[C_2_C_1_im]Cl	synthetic urine	100	IL + K_3_PO_4_	[129]
EE2	[C_4_C_1_im][N(CN)_2_]	standard	1000	IL + KNaC_4_H_4_O_6_	[126]
Quinine	[C_4_C_1_im]Cl	human plasma	N.R.*^a^*	IL + K_2_HPO_4_	[134]
Caffeine	[N_4444_]Cl	pretreated wastewater effluent	28,595 *^b^*	IL + K_3_C_6_H_5_O_7_	[127]
Carbamazepine	[N_4444_]Cl	pretreated wastewater effluent	8259 *^b^*	IL + K_3_C_6_H_5_O_7_	[127]
Ciprofloxacin	[N_4444_]Cl	pretreated wastewater effluent	1000	IL + K_3_C_6_H_5_O_7_	[135]
Diclofenac	[N_4444_]Cl	pretreated wastewater effluent	1000	IL + K_3_C_6_H_5_O_7_	[135]
	[N_4444_]Cl	environmental water samples	1230.8	IL + Na_2_C_4_H_4_O_5_	[140]
Codeine	[C_4_C_1_im]Cl	*Pericarpium papaveris*	N.R.*^a^*	IL + K_2_HPO_4_	[136]
Papaverine	[C_4_C_1_im]Cl	*Pericarpium papaveris*	N.R.*^a^*	IL + K_2_HPO_4_	[136]
Azithromycin	[C_4_C_1_im][BF_4_]	Environmental water samples	N.R.*^a^*	IL + Na_2_CO_3_	[137]
Mydecamycin	[C_4_C_1_im][BF_4_]	Environmental water samples	N.R.*^a^*	IL + NaH_2_PO_4_	[137]
Acetylspiramycin	[C_4_C_1_im][BF_4_]	Environmental water samples	10	IL + NaH_2_PO_4_	[138]
Chloramphenicol	[C_4_C_1_im][BF_4_]	Honey, milk and water samples	22.5	IL + Na_3_C_6_H_5_O_7_	[139]
	[N_4444_]Cl	Environmental water samples	1216	IL + Na_2_C_4_H_4_O_5_	[140]
	[C_1_C_1_C_1_C_1_guan][TEMPO-OSO_3_]	Environmental water samples	147.2	IL + K_3_PO_4_	[145]
Indomethacin	[N_4444_]Cl	Environmental water samples	1238	IL + Na_2_C_4_H_4_O_5_	[140]
Ibuprofen	[N_4444_]Cl	Environmental water samples	1228	IL + Na_2_C_4_H_4_O_5_	[140]
Ketoprofen	[N_4444_]Cl	Environmental water samples	1230	IL + Na_2_C_4_H_4_O_5_	[140]
Flurbiprofen	[N_4444_]Cl	Environmental water samples	1218	IL + Na_2_C_4_H_4_O_5_	[140]
Acethylcholinesterase inhibitors	[N_111(2OH)_][Sac]	Tablet and human urine	153	IL + Na_2_CO_3_	[141]
Tetracycline	[aC_1_im]Cl	Milk and honey	N.R.*^a^*	IL + K_2_HPO_4_ + Triton X-100	[142]
Sulfonamides	[C_6_C_1_im]Cl	Human plasma	N.R.*^a^*	IL + K_2_HPO_4_ + SDS	[143]
Berberine hydrochloride	[N_115(2OH)_][TEMPO-OSO_3_]	*Rhizoma coptidis*	127.68	IL + K_3_PO_4_	[146]
IL-DLLME					
BPA	[C_6_C_1_im][FeCl_4_]	Vegetable oils	N.R.*^a^*	Extraction Solvent: IL Dispersant: acetone Magnetic aid: Fe_3_O_4_	[149]
4-nonylphenol	[C_6_C_1_im][FeCl_4_]	Vegetable oils	N.R.*^a^*	Extraction solvent: IL Dispersant: acetone Magnetic aid: Fe_3_O_4_	[149]
Triclosan	[C_4_C_1_im][BF_4_] + [C_4_C_1_im][NPA]	Human serum and urine	N.R.*^a^*	Extraction solvent: [C_8_C_1_im][PF_6_] Dispersant: ([C_4_C_1_im][BF_4_] + [C_4_C_1_im][NPA]) Ion exchange reagent: NH_4_PF_6_	[150]
Methyltriclosan	[C_4_C_1_im][BF_4_] + [C_4_C_1_im][NPA]	Human serum and urine	N.R.*^a^*	Extraction solvent: [C_8_C_1_im][PF_6_] Dispersant: ([C_4_C_1_im][BF_4_] + [C_4_C_1_im][NPA]) Ion exchange reagent: NH_4_PF_6_	[150]
Tetracycline	[C_2_C_1_im][BF_4_] + [C_4_C_1_im][NPA]	Milk and eggs	N.R.*^a^*	Extraction solvent: [C_6_C_1_im][PF_6_] Dispersant: ([C_4_C_1_im][BF_4_] + [C_4_C_1_im][NPA]) Ion exchange reagent: NH_4_PF_6_	[151]
Oxytetracycline	[C_2_C_1_im][BF_4_] + [C_4_C_1_im][NPA]	Milk and eggs	N.R.*^a^*	Extraction solvent: [C_6_C_1_im][PF_6_] Dispersant: ([C_4_C_1_im][BF_4_] + [C_4_C_1_im][NPA]) Ion exchange reagent: NH_4_PF_6_	[151]
Chlorotetracycline	[C_2_C_1_im][BF_4_] + [C_4_C_1_im][NPA]	Milk and eggs	N.R.*^a^*	Extraction solvent: [C_6_C_1_im][PF_6_] Dispersant: ([C_4_C_1_im][BF_4_] + [C_4_C_1_im][NPA]) Ion exchange reagent: NH_4_PF_6_	[151]
Doxycycline	[C_2_C_1_im][BF_4_] + [C_4_C_1_im][NPA]	Milk and eggs	N.R.*^a^*	Extraction solvent: [C_6_C_1_im][PF_6_] Dispersant: ([C_4_C_1_im][BF_4_] + [C_4_C_1_im][NPA]) Ion exchange reagent: NH_4_PF_6_	[151]
in situ IL-DLLME					
Tetracycline	[C_7_H_7_C_1_im]Cl	Milk, honey, egg	25	Extraction solvent: IL Ion exchange reagent: NH_4_PF_6_	[152]
Methacycline	[C_7_H_7_C_1_im]Cl	Milk, honey, egg	98	Extraction solvent: IL Ion exchange reagent: NH_4_PF_6_	[152]
Chlortetracycline	[C_7_H_7_C_1_im]Cl	Milk, honey, egg	60	Extraction solvent: IL Ion exchange reagent: NH_4_PF_6_	[152]
Doxycycline	[C_7_H_7_C_1_im]Cl	Milk, honey, egg	56	Extraction solvent: IL Ion exchange reagent: NH_4_PF_6_	[152]
BPA	[C_4_C_1_im]Cl	Environmental water samples and effluents	130–149	Extraction solvent: IL Ion exchange reagent: LiNTf_2_	[153]
	[C_8_C_1_im]Cl	Toys and pacifiers	299	Extraction solvent: IL Non-stick agent: X-100 Ion exchange reagent: LiNTf_2_	[154]
4-cumylphenol	[C_4_C_1_im]Cl	Environmental water samples and effluents	965–1037	Extraction solvent: IL Ion exchange reagent: LiNTf_2_	[153]
4-*tert*-Butylphenol	[C_4_C_1_im]Cl	Environmental water samples and effluents	354–410	Extraction solvent: IL Ion exchange reagent: LiNTf_2_	[153]
4-Octylphenol	[C_4_C_1_im]Cl	Environmental water samples and effluents	402–463	Extraction solvent: IL Ion exchange reagent: LiNTf_2_	[153]
4-*tert*-Octylphenol	[C_4_C_1_im]Cl	Environmental water samples and effluents	891–967	Extraction solvent: IL Ion exchange reagent: LiNTf_2_	[153]
4-*n*-Nonylphenol	[C_4_C_1_im]Cl	Environmental water samples and effluents	682–762	Extraction solvent: IL Ion exchange reagent: LiNTf_2_	[153]
Myclobutanil	[C_4_C_4_im]Br	Environmental water samples	323	Extraction solvent: IL Ion exchange reagent: LiNTf_2_ Magnetic aid: Fe_3_O_4_	[155]
Tebuconazole	[C_4_C_4_im]Br	Environmental water samples	211	Extraction solvent: IL Ion exchange reagent: LiNTf_2_ Magnetic aid: Fe_3_O_4_	[155]
Cyproconazole	[C_4_C_4_im]Br	Environmental water samples	187	Extraction solvent: IL Ion exchange reagent: LiNTf_2_ Magnetic aid: Fe_3_O_4_	[155]
Prothioconazole	[C_4_C_4_im]Br	Environmental water samples	247	Extraction solvent: IL Ion exchange reagent: LiNTf_2_ Magnetic aid: Fe_3_O_4_	[155]
Sulfamethazine	[C_4_C_1_im-TEMPO]Cl	Milk	44.3	Extraction solvent: IL Ion exchange reagent: KPF_6_	[156]
Sulfamonomethoxine	[C_4_C_1_im-TEMPO]Cl	Milk	47.0	Extraction solvent: IL Ion exchange reagent: KPF_6_	[156]
Sulfadiazine	[C_4_C_1_im-TEMPO]Cl	Milk	46.5	Extraction solvent: IL Ion exchange reagent: KPF_6_	[156]
Sulfamerazine	[C_4_C_1_im-TEMPO]Cl	Milk	42.4	Extraction solvent: IL Ion exchange reagent: KPF_6_	[156]
Sulfamethizole	[C_4_C_1_im-TEMPO]Cl	Milk	43.9	Extraction solvent: IL Ion exchange reagent: KPF_6_	[156]

*^a^* N.R.—Not reported. *^b^* Theoretically expected values.

**Table 4:** Application of hydrophilic ILs in the recovery and purification of pharmaceuticals.

Pharmaceutical	Hydrophilic IL	Pharmaceutical Source	Recovery/Purification Method	Operating Conditions		Ref.
				T (K)	System Components	
Tetracycline	[N_111(2OH)_]Cl	standard	ABS	298	IL + K_3_PO_4_	[40]
	[N_111(2OH)_][Ace]	standard	ABS	298	IL + K_3_PO_4_	[40]
	[N_111(2OH)_][Lev]	standard	ABS	298	IL +K_3_PO_4_	[40]
	[N_111(2OH)_][Glt]	standard	ABS	298	IL + K_3_PO_4_	[40]
	[N_111(2OH)_][Suc]	standard	ABS	298	IL + K_3_PO_4_	[40]
	[N_111(2OH)_]Cl	fermentation broth	ABS	298	IL + K_3_PO_4_	[39]
	[N_111(2OH)_][Bic]	fermentation broth	ABS	298	IL + K_3_PO_4_	[39]
Ciprofloxacin (and its hydrochloride salt)	[C_4_C_1_im][CF_3_SO_3_]	standard	ABS	298	IL + lysine	[177]
Ibuprofen	[C_4_C_1_im]Cl	pills	TPP + precipitation with antisolvent	298	IL + K_3_C_6_H_5_O_7_/C_6_H_8_O_7_ (pH = 7)	[180]
	[P_4441_][C_1_SO_4_]	standard	ABS + precipitation with antisolvent	298	IL + Al_2_(SO_4_)_3_	[163]
	[N_111(2OH)_]Cl	standard	ABS	298, 333	IL + Tween 80	[176]
	[C_4_C_1_im][SCN]	standard	Crystallization with antisolvent	---	IL aqueous solution	[59]
Naproxen	[C_4_C_1_im]Cl	pills	TPP + precipitation with antisolvent	298	IL + K_3_C_6_H_5_O_7_/C_6_H_8_O_7_ (pH = 7)	[180]
	[P_4441_][C_1_SO_4_]	standard	ABS + precipitation with antisolvent	298	IL + Al_2_(SO_4_)_3_	[163]
Ketoprofen	[C_4_C_1_im]Cl	pills	TPP + precipitation with antisolvent	298	IL + K_3_C_6_H_5_O_7_/C_6_H_8_O_7_ (pH = 7)	[180]
	[P_4441_][C_1_SO_4_]	standard	ABS + precipitation with antisolvent	298	IL + Al_2_(SO_4_)_3_	[163]
Paracetamol	[N_2222_]Br	pills	ABS	298	IL + K_3_C_6_H_5_O_7_/C_6_H_8_O_7_ (pH = 7) or K_2_CO_3_	[167]
	[N_4444_]Br	pills	ABS	298	IL + K_3_C_6_H_5_O_7_/C_6_H_8_O_7_ (pH = 7) or K_2_CO_3_	[167]
	[C_4_C_1_im]Cl	pills	ABS +ABS	298	IL + Pluronic PE 6200	[175]
	[C_2_C_1_im][C_1_CO_2_]*_x_*[NTf_2_]_1-*x*_	standard	Crystallization with anti-solvent	298	IL	[185]
Diclofenac	[P_4441_][C_1_SO_4_]	standard	ABS + precipitation with antisolvent	298	IL + Al_2_(SO_4_)_3_	[163]
	[N_111(2OH)_]Cl	standard	ABS	298, 333	IL + Tween 80	[176]
Fluoxetine hydrochloride	[C_2_C_1_im][C_1_CO_2_]	standard	ABS	298	IL + 1,3-dioxolane	[178]
Paroxetine hydrochloride	[C_2_C_1_im][C_1_CO_2_]	standard	ABS	298	IL + 1,3-dioxolane	[178]
Sertraline hydrochloride	[C_2_C_1_im][C_1_CO_2_]	standard	ABS	298	IL + 1,3-dioxolane	[178]
17β-estradiol	([N_000(2OH)_][C_1_CO_2_] + [N_00(2OH)(2OH)_][C_1_CO_2_]), ([N_000(2OH)_][C_1_CO_2_] + [N_00(2OH)(2OH)_][C_3_CO_2_])	standard	ABS	298	IL mixture + acetonitrile	[179]
estriol	([N_000(2OH)_][C_1_CO_2_] + [N_00(2OH)(2OH)_][C_1_CO_2_]), ([N_000(2OH)_][C_1_CO_2_] + [N_00(2OH)(2OH)_][C_3_CO_2_])	standard	ABS	298	IL mixture + acetonitrile	[179]
EE2	([N_000(2OH)_][C_1_CO_2_] + [N_00(2OH)(2OH)_][C_1_CO_2_]), ([N_000(2OH)_][C_1_CO_2_] + [N_00(2OH)(2OH)_][C_3_CO_2_])	standard	ABS	298	IL mixture + acetonitrile	[179]
progesterone	([N_000(2OH)_][C_1_CO_2_] + [N_00(2OH)(2OH)_][C_1_CO_2_]), ([N_000(2OH)_][C_1_CO_2_] + [N_00(2OH)(2OH)_][C_3_CO_2_])	standard	ABS	298	IL mixture + acetonitrile	[179]
Intermediate amine of the aliskiren synthesis	[C_2_C_1_im][C_1_CO_2_]	Reaction mixtures	LLE	294	IL + ethyl acetate	[183]
methyl-(Z)-α-acetamido cinnamate	[C_4_C_1_im][BF_4_]	standard	Crystallization with anti-solvent or thermal shift	278–338	IL + CO_2_	[184]

## Data Availability

Data are contained within this manuscript.

## Abbreviations

[C*_n_*Car]^+^	Carnitine alkyl ester
[C*_n_*C_1_C_1_im]^+^	1-alkyl-2,3-dimethylimidazolium
[C*_n_*C_1_im]^+^	1-alkyl-3-methylimidazolium
[C*_n_*C_1_im]^+^	1-alkyl-3-butylimidazolium
[C*_n_*C_1_pip]^+^	1-alkyl-1-methylpiperidinium
[C*_n_*C_1_pyr]^+^	1-alkyl-1-methylpyrrolidinium
[C*_n_*NH_3_]^+^	*N*-alkylammonium
[C_1_C_1_C_1_C_1_guan]^+^	1,1,3,3-tetramethylguanidinium
[C_4_(C_n_im)_2_]^2+^	1,1’-(butane-1,4-diyl)bis(3-alkylimidazolium)
[C_7_H_7_C_1_im]^+^	1-benzyl-3-methylimidazolium
[N*_nnnn_*]^+^	tetraalkylammonium
[N_000(2OH)_]^+^	2-hydroxyethylammonium
[N_00(2OH)(2OH)_]^+^	bis(2-hydroxyethyl) ammonium
[N_11*n*(2OH)_]^+^	*N*-Alkyl-*N*,*N*-dimethyl-*N*-(2-hydroxyethyl)ammonium
[N_111(2OH)_]^+^	*N*,*N*,*N*-trimethyl-*N*-(2-hydroxyethyl)ammonium (cholinium)
[P*_nnnn_*]^+^	Tetraalkylphosphonium
[P*_nnn_*_1_]^+^	Trialkylmethylphosphonium
[P_666,14_]^+^	Trihexyltetradecyl phosphonium
[OHC*_n_*C_1_im]^+^	1-hydroxyalkyl-3-methylimidazolium
[BF_4_]^–^	Tetrafluoroborate
[Bic]^–^	Bicarbonate
[CF_3_CO_2_]^–^	Trifluoroacetate
[CF_3_SO_3_]^–^	Trifluoromethanesulfonate
[C*_n_*CO_2_]^–^	Alkylcarboxylate
[C_1_SO_4_]^–^	Methylsulfate
[C_1_SO_3_]^–^	Methylsulfonate
[Gal]^–^	Gallate
[Ger]^–^	Geranate
[Glt]^–^	Glutarate
[Ibu]^–^	Ibuprofenate
[Lev]^–^	Levulinate
[NPA]^–^	Naphthoate
[NTf_2_]^–^	Bis(trifluoromethylsulfonyl)amide
[N(CN)_2_]^–^	Dicyanamide
[Ole]^–^	Oleate
[PF_6_]^–^	Hexafluorophosphate
[Sal]^–^	Salicylate
[SCN]^–^	Thiocyanate
[Suc]^–^	Succinate
[TEMPO-OSO_3_]^–^	2,2,6,6-tetramethyl-1-piperidinyloxyl-4-sulfate
[TsO]^–^	Tosylate
[Trp]^–^	Triptophanate
[Van]^–^	Vanillate
[(CH_3_O)_2_PO_2_]^–^	Dimethyl phosphate
ABS	Aqueous biphasic systems
API	Active pharmaceutical ingredient
BPA	Bisphenol A
Br^–^	Bromide
CAF	Caffeine
CBZ	Carbamazepine
CE	Capillary electrophoresis
CF	Concentration factor
Cl^–^	Chloride
CMC	Critical micelle concentration
DAD	Diode array detector
DLLME	Dispersive liquid-liquid microextraction
EC_50_	The effective concentration resulting in a 50% reduction of processes
EE2	17α-ethinylestradiol
FD	Fluorescence detection
FESEM	Field emission scanning microscopy
FQ	Fluoroquinolones
GC	Gas chromatography
HPLC	High performance liquid chromatography
IL	Ionic liquid
LC	Liquid chromatography
LLE	Liquid-liquid extraction
MS	Mass spectrometry
NMR	Nuclear magnetic resonance spectroscopy
NSAID	Non-steroidal anti-inflammatory drug
OECD	Organization for Economic Cooperation and Development
PEG	Polyethylene glycol
PIL	Polymerizable biobased ionic liquid
PPG	Polypropylene glycol
SAIL	Surface active ionic liquids
SPE	Solid-phase extraction
TL	Tie-line
TPP	Three-phase partitioning
TSIL	Task-specific ionic liquid
UV	Ultraviolet detector
VOC	Volatile organic compound
WWTP	Wastewater treatment plant
